# A defined community of core gut microbiota members promotes cognitive performance in honey bees

**DOI:** 10.1073/pnas.2608600123

**Published:** 2026-05-20

**Authors:** Amélie Cabirol, Andrew Quinn, Julie Schafer, Nicolas Neuschwander, Lucie Kesner, Joanito Liberti, Philipp Engel

**Affiliations:** ^a^Department of Fundamental Microbiology, University of Lausanne, Lausanne 1015, Switzerland; ^b^Department of Ecology and Evolution, University of Lausanne, Lausanne 1015, Switzerland

**Keywords:** learning, memory, gut bacteria, symbiosis, *Apis mellifera*

## Abstract

Gut microbiota across animals have been shown to influence host cognition and behavior. However, it remains unclear whether these cognitive effects are driven by specific bacterial species or arise from community-level interactions. Here, we leveraged the honey bee (*Apis mellifera*) as a model system, which harbors a simple and well-characterized gut microbiota that is experimentally tractable and has been previously shown to impact host cognition. We established a defined bacterial community—composed of core members of the honey bee gut microbiota. Gnotobiotic bee experiments with the full community, communities missing individual members, or individual members showed that only the full community enhanced honey bees’ performances in odor discrimination learning and short-term memory compared to microbiota-deprived bees. Metabolomic analyses identified several metabolites associated with learning success that mapped to pathways modulated by microbial colonization, including tryptophan metabolism, nucleoside metabolism, and lysine degradation. However, many of these metabolites were not altered by removing individual members from the full microbial community. This suggests that microbiota-mediated improvements in cognition are emergent properties of the community as a whole, rather than the result of individual metabolites or specific bacterial taxa acting alone. Our findings support a systems-level view of the microbiome, suggesting that understanding and manipulating host development, particularly in relation to brain function, should prioritize microbial community function (e.g., metabolic pathways) over taxonomic composition alone.

## Significance

Gut microbiota have been implicated in shaping host cognition, yet the mechanisms through which microbial communities influence brain function remain poorly understood. Using the honey bee, we demonstrate that cognitive benefits are not conferred by individual bacterial genera or partially assembled communities, but require the full complement of core gut microbes. Metabolomic profiling revealed that key pathways linked to learning are only significantly modulated in the presence of the complete microbial community. This shows that cognitive enhancement is an emergent property of the microbial ecosystem rather than the product of specific taxa or metabolites. Our results underscore the importance of community-level interactions in microbiota–brain communication, with broad implications for the design of microbiome-based therapies targeting neurodevelopment and cognitive function.

Over the past decade, accumulating evidence has highlighted the critical role of the gut microbiota in modulating cognitive performance (1, 2). Distinct gut microbiota profiles have been linked to cognitive impairments in humans, while studies in germ-free animals have demonstrated the profound impact of microbiota absence on cognitive function (3–7). Although several studies have begun to uncover mechanisms through which individual bacterial strains influence host cognition, the specific contributions of individual members in a complex community to cognitive processes remain poorly understood.

The honey bee (*Apis mellifera*) is emerging as a useful model to unravel the proximate mechanisms underlying the effect of the gut microbiota on complex cognitive and behavioral phenotypes (8–10). Microbiota-deprived (MD) honey bees can be obtained by extracting mature pupae from their wax cell and letting them develop into adults in a sterilized environment (11). These bees typically lack the specific bacteria of the honey bee gut microbiota which comprises 8 to 10 bacterial genera. Members of these genera can be cultured in the laboratory and subsequently introduced into MD bees. This approach enables the generation of gnotobiotic bees harboring a gut microbial community of defined composition (12, 13). Pavlovian associative learning, an evolutionary conserved cognitive process, has been extensively studied in this insect thanks to the development of the olfactory conditioning of the proboscis extension response (PER) more than 60 y ago (14, 15). In this conditioning protocol, harnessed bees are trained to associate an odor with a sucrose reward whose presentation to the bee’s antennae automatically triggers the PER. Once the odor–food association is learned, bees respond with proboscis extension to the conditioned odor. Using this protocol, two recent studies showed that MD bees display memory deficits compared to bees harboring the full native gut microbiota (9, 16). Moreover, monoinoculation with *Gilliamella* or *Lactobacillus* sp., two core members of the bee gut microbiota, partially restored cognitive performance under specific dietary conditions. Although these results provide initial mechanistic insights into the gut–brain axis in bees, the relative contributions of specific bacterial taxa vs. microbial communities to the observed cognitive improvements, and the associated metabolic changes in the gut, have not been systematically assessed.

**Fig. 1.**
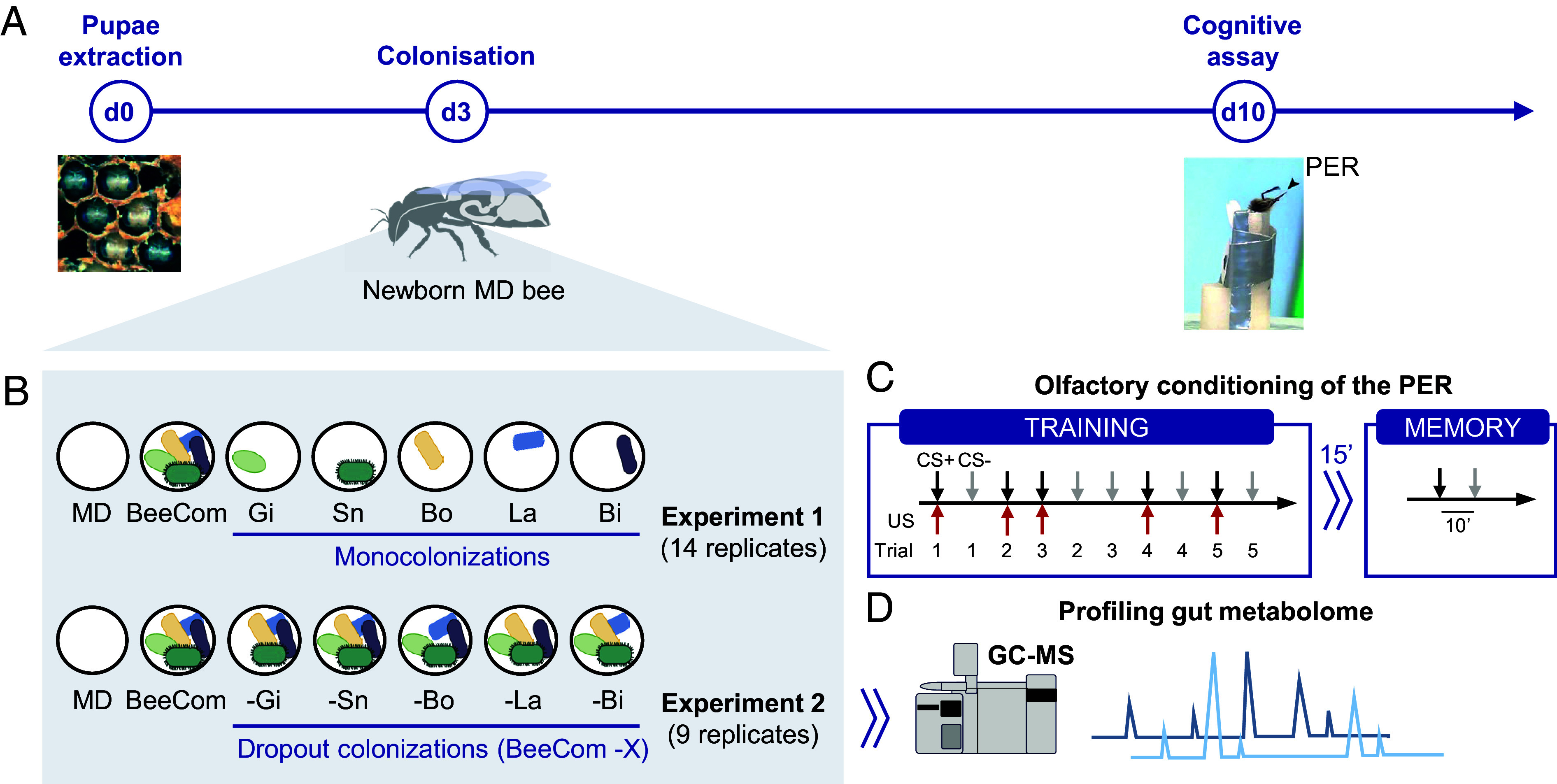
Experimental design for cognitive assays on gnotobiotic bees. (*A*) Timeline of the experiments. Honeybee pupae were extracted from their cell and placed in a sterilized laboratory environment. Three days later, newborn MD bees were fed a defined bacterial inoculum, the composition of which varied depending on the gnotobiotic group. Gnotobiotic bees were kept in separate cages of approximately 15 individuals until the cognitive assay which consisted in a pavlovian conditioning of the PER (black arrow). Guts were sampled after the memory test to quantify bacterial loads and analyze the metabolic content. (*B*) Two different experiments were carried out. In experiment 1 (monocolonizations), MD bees were compared to bees colonized with the full BeeCom or individual genera. In experiment 2 (dropout colonizations), MD bees were compared to bees colonized with the full BeeCom or the BeeCom depleted of one genus at a time. The BeeCom consists of 11 strains of the major bee gut genera *Gilliamella* (Gi), *Snodgrassella* (Sn), *Bombilactobacillus* (Bo), *Lactobacillus* (La), *Bifidobacterium* (Bi). (*C*) During the olfactory conditioning of the PER, bees were trained to discriminate a rewarded odor (conditioned stimulus, CS+) from an unrewarded odor (CS−) within five presentations of each. The reward consisted in sucrose solution (unconditioned stimulus, US). The order of presentation of the CS during the training and memory test was randomized between experimental replicates. Short-term memory was assessed 15 min after conditioning by presenting both CS. (*D*) Gut samples from experiment 2 were subjected to GC–MS-based metabolomic analysis to assess changes in gut metabolic profiles across different treatments and to correlate these changes with the cognitive performance of individual bees.

Here, we assessed the importance of different bacterial genera in a defined bacterial community, hereafter called the BeeCom, for bees’ cognitive abilities (Fig. 1). The BeeCom was composed of bacteria of the five most prevalent and abundant genera detected in the gut of adult worker bees: *Gilliamella*, *Snodgrassella*, *Bombilactobacillus*, *Lactobacillus*, and *Bifidobacterium* (11, 17). MD bees were inoculated with different subsets of the BeeCom, and their associative learning and memory performance was assessed using a Pavlovian conditioning assay. In the first experiment (monocolonizations), we tested which bacterial genera, if any, are sufficient to confer the beneficial effect of the BeeCom on host cognitive performance. In the second experiment (dropouts), we assessed which bacterial genera are essential for this effect, and whether metabolite abundances in the gut correlate with learning performance. The monocolonization experiment showed that the BeeCom significantly improved bees’ olfactory learning and memory performances while none of the community members alone could recapitulate those effects. In the dropout experiment, excluding any member of the BeeCom resulted in performance similar to MD bees. Larger and significant effects were observed when excluding Gilliamella or Snodgrassella from the BeeCom. Metabolites positively correlated with learning performance were modulated by multiple members of the BeeCom. Together, these results indicate that the cognitive benefit is an additive or emergent property of the BeeCom community.

## Results

### The BeeCom, but Not Its Single Members, Supports Olfactory Learning and Memory.

We first compared associative learning and memory performances of MD bees, bees colonized with the BeeCom and bees colonized with single BeeCom members (i.e., monocolonization experiment). The gut bacterial composition of each gnotobiotic group was validated by qPCR (*SI Appendix*, Fig. S1). MD bees had low bacterial loads in the gut and lacked the specific bacteria of the bee gut microbiota. The relatively high background signal detected with universal primers has previously been explained by the amplification of chloroplast and host mitochondria DNA, and sometimes low levels of environmental bacteria (10). Most community members showed consistent colonization when inoculated with the BeeCom, except for *Gilliamella* whose colonization success varied between experimental replicates. The inoculated bacteria did not affect the mortality in cages, the weight of individual bees (without gut), or the sucrose consumption per bee (*SI Appendix*, Fig. S2). Bee responsiveness to an ascending concentration series of sucrose solution was measured to discriminate the effects of gut bacteria on sucrose perception from effects on learning and memory per se (18). It did not differ between the gnotobiotic groups (*SI Appendix*, Fig. S3) and did not correlate with learning performance (Spearman correlation test; rho = −0.045; *P* = 0.62).

**Fig. 2.**
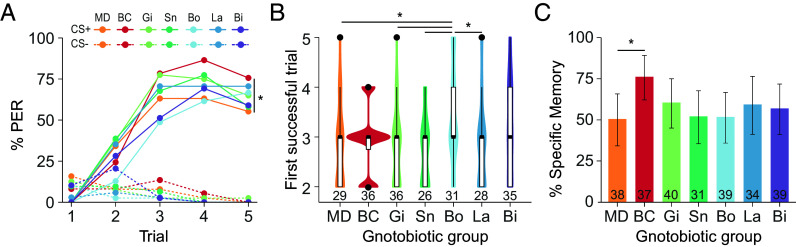
The BeeCom, but not its single members, facilitated olfactory learning and memory performances. (*A*) Learning performance of MD bees, bees colonized with the defined community BeeCom (BC), and bees monocolonized with *Gilliamella* (Gi), *Snodgrassella* (Sn), *Bombilactobacillus* (Bo), *Lactobacillus Firm-5* (La), or *Bifidobacterium* (Bi). The percentage of bees showing a PER to the rewarded (CS+; plain lines) and unrewarded (CS−; dashed lines) odor is displayed for each gnotobiotic group across the five learning trials [GLMM; Post hoc comparisons in trial 5; *P* < 0.05 (*)]. (*B*) Learning speed reflected by the first trial in which individual bees respond to the rewarded odor [Dunn’s tests; *P* < 0.05 (*)]. (*C*) Percentage of gnotobiotic bees showing PER to the rewarded but not to the unrewarded odor during the memory test [GLM; *P* < 0.05 (*)]. Error bars show the bootstrapped 95% CI. Sample sizes are indicated at the bottom of the plots (*B* and *C*) and are identical for panels (*A*) and (*C*). They correspond to the total number of bees tested across the 14 experimental replicates.

Using a differential olfactory conditioning of the PER, bees were trained to discriminate two odors across five learning trials (Fig. 1*C*). One odor was learned through its association with a sucrose reward (conditioned stimulus, CS+) and a second odor through its association with the absence of reward (CS−). The bees received five presentations of each odor in a randomized order. All seven groups of gnotobiotic bees solved the differential learning task (Fig. 2*A*). Bees showed progressively more PER to the CS+ compared to the CS− (generalized linear mixed-effects model, GLMM; CS × Trial: *P* < 0.0001), resulting in higher responses to the CS+ in the final trial for all groups [post hoc pairwise comparisons corrected with the false discovery rate (FDR) procedure; *P* < 0.0001]. However, the learning performance of bees colonized with the BeeCom was significantly better than that of MD bees (GLMM; Group × Trial: estimates ± SE = 0.48 ± 0.23, *P* < 0.05). They exhibited a 20% higher PER to the CS+ in the final learning trial (FDR correction; *P* < 0.05).

In contrast, monocolonized bees showed intermediate learning performances as their responses to the CS+ were not significantly different from MD and BeeCom-colonized bees in the final trial (FDR correction; *P* > 0.05). Furthermore, *Bombilactobacillus* monocolonization led to a significantly slower learning speed compared to MD bees (GLMM; Group × Trial: estimate ± SE = 0.44 ± 0.22, *P* < 0.05) (Fig. 2*B*; Dunn test with FDR correction, *P* < 0.05).

Consistent with the learning performance, colonization with the BeeCom improved short-term memory (Fig. 2*C*). The percentage of BeeCom-colonized bees responding to the CS+ but not to the CS− (i.e., showing specific memory) was significantly higher than that of MD bees (GLM: estimates ± SE = 1.16 ± 0.50, *P* < 0.05). None of the other groups differed significantly from the MD group (GLM, *P* > 0.05).

In summary, these results show that colonization with a defined community consisting of bee gut microbiota core genera enhances both learning and short-term memory abilities of honey bees relative to MD bees. Monocolonization with individual core genera showed intermediate effects on learning performance, indicating that individual bacterial genera can modulate learning behavior but cannot fully replicate the effect of the complete microbial community.

### All Bacterial Genera Participate in the BeeCom-Associated Cognitive Facilitation.

As no single genera provided the same benefit as the BeeCom, we next generated dropout communities by selectively excluding single genera from the BeeCom and thereby assessed the importance of each genus of the community for learning and memory performances (Fig. 1). The gnotobiotic status of the seven groups was confirmed after the cognitive assays by qPCR on gut samples (*SI Appendix*, Fig. S1). Again, *Gilliamella* colonized with variable success when inoculated in a community with other genera.

**Fig. 3.**
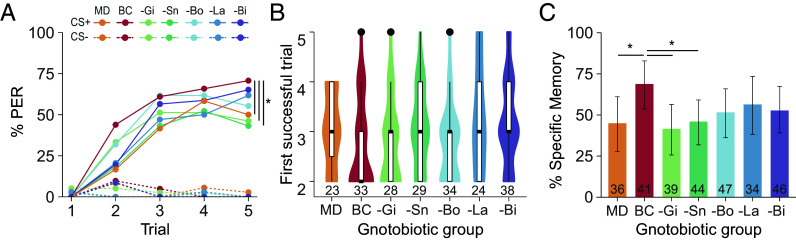
All members of the BeeCom support olfactory learning and memory, but to different degrees. (*A*) Learning performance of MD bees, bees colonized with the defined community BeeCom (BC), and bees colonized with dropout communities lacking *Gilliamella* (-Gi), *Snodgrassella* (-Sn), *Bombilactobacillus* (-Bo), *Lactobacillus* (-La), or *Bifidobacterium* (-Bi). The percentage of bees showing a PER to the rewarded (CS+; plain lines) and unrewarded (CS−; dashed lines) odor is displayed for each gnotobiotic group across the five learning trials [GLMM; Post hoc comparisons in trial 5; *P* < 0.05 (*)]. (*B*) Learning speed reflected by the first trial in which individual bees respond to the rewarded odor [Dunn’s tests; *P* < 0.05 (*)]. (*C*) Percentage of gnotobiotic bees responding to the CS+ but not to the CS− during the memory test [GLM; *P* < 0.05 (*)]. Error bars show the bootstrapped 95% CI. Sample sizes are indicated at the bottom of the plots (*B* and *C*) and are identical for panels (*A*) and (*C*). They correspond to the total number of bees tested across the nine experimental replicates.

All groups discriminated the CS+ from the CS− across the five learning trials (Fig. 3*A*; GLMM; *CS* × *Trial*: *P* < 0.0001; FDR correction *P* < 0.0001 in the last trial for all groups). This second series of experiments confirmed that the proportion of bees that learned the CS+ was 20% higher in the BeeCom-colonized group than in MD bees. (FDR correction: *P* < 0.05 in the last trial). Among the dropout groups, only the absence of *Gilliamella* and *Snodgrassella* led to a statistically significant reduction of responses to the CS+ in the last trial compared to the BeeCom group (FDR correction: *P* < 0.005). However, none of the dropout treatments differed significantly from the MD group (FDR correction, *P* > 0.05 for all pairwise comparisons), suggesting that all community members contribute collectively to the associative learning ability of the host. Unlike the previous monocolonization experiment, in which monocolonization with *Bombilactobacillus* showed slower learning than other treatments, learning speed was not affected by the absence of single BeeCom members (Fig. 3*B*; Kruskall-Wallis, *P* = 0.063).

Short-term memory was reduced in MD bees (GLM: estimates ± SE = −1.11 ± 0.48, *P* < 0.05) as well as in bees colonized with the dropout communities lacking *Gilliamella* (GLM: estimates ± SE = −1.24 ± 0.48, *P* < 0.01) or *Snodgrassella* (GLM: estimates ± SE = −0.97 ± 0.46, *P* < 0.05) compared to BeeCom-colonized bees (Fig. 3*C*). Again, the dropout experiment showed that only the BeeCom increased learning and memory performances compared to MD bees suggesting additive or synergistic effects of community members.

### Metabolic Functions Emerging from the BeeCom Associate with Learning Success.

**Fig. 4.**
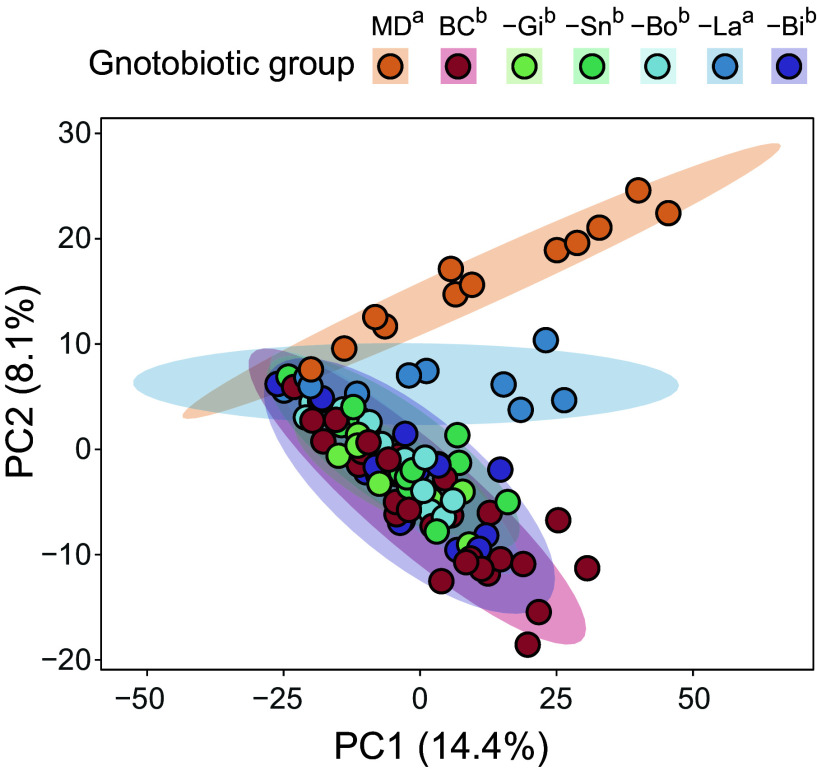
Gut metabolic profiles are partly explained by the gnotobiotic group. PCA of 582 metabolic features detected by GC–MS in the gut of MD (n = 12) bees, bees colonized with the full BeeCom (BC; n = 33) and bees colonized with the BeeCom lacking *Gilliamella* (-Gi; n = 12), *Snodgrassella* (-Sn; n = 14), *Bombilactobacillus* (-Bo; n = 12), *Lactobacillus* (-La; n = 10), or *Bifidobacterium* (-Bi; n = 14). Different letters next to the gnotobiotic group indicate significant differences (pairwise PERMANOVA; *P* < 0.05).

To identify if bacterial metabolism correlates with enhancement in bee olfactory learning and memory performances, 582 features in the gut of gnotobiotic bees from the dropout experiment were measured by GC–MS (Fig. 4 and *SI Appendix*, Figs. S4 and S6). The gnotobiotic group explained 18% of the metabolic profile variance in a principal component analysis (PCA) (Fig. 4; PERMANOVA: F = 3.72, R^2^ = 0.18, *P* = 0.001). BeeCom-colonized bees and MD bees clustered separately, (pairwise PERMANOVA: *P* < 0.05), while removing *Lactobacillus* from the BeeCom induced a significant metabolic shift away from BeeCom*-*colonized bees (*P* < 0.05) and toward MD bees (*P* = 0.11). In contrast, all other dropout groups clustered closely with BeeCom-colonized bees. These results indicate that the genus *Lactobacillus* has the strongest effect on the detected metabolite changes in the bee gut.

Nearly one third of the metabolic features (177 of 582) differed in abundance between BeeCom-colonized bees and at least one other gnotobiotic group (*SI Appendix*, Fig. S4 and Dataset S1). Among the dropout communities, the number of features that differed significantly from the BeeCom group ranged from as few as 3 in the *Gilliamella* dropout to as many as 63 features in the *Lactobacillus* dropout. As expected, MD bees differed the most, with 151 significantly altered features, including 82 that did not significantly differ in any dropout condition. This highlights the prevalence of metabolic redundancy in the gut community, where multiple bacterial genera produce or deplete the same compound.

Our cognitive assay revealed that short-term memory performance was impaired in dropout groups lacking *Gilliamella* or *Snodgrassella*. However, these two conditions did not share any differentially abundant metabolic features (*SI Appendix*, Fig. S4). The three features that were significantly less abundant in the absence of *Gilliamella* included sucrose, galactinol, and one undefined compound. In contrast, the *Snodgrassella* dropout resulted in more pronounced metabolic changes, characterized by higher abundances of 3-hydroxy-3-methylglutaric acid and homoserine, and lower abundances of several amino acids and their catabolites, including alanine, anthranilic acid, 3-hydroxyanthranilic acid, tyramine, and 5-hydroxylysine.

As all gnotobiotic groups contained bees that passed and failed the short-term memory test, we then assessed the specific association of gut metabolites with memory performance across all gnotobiotic groups. No metabolites were significantly associated with the performance in the memory test (pairwise Wilcoxon tests corrected with Benjamini–Hochberg method; *P* > 0.05 for all detected metabolites), and a random forest model using metabolite abundances to predict success in the test performed no better than random chance (Accuracy: 0.55, 95% CI [0.32, 0.77], *P* > 0.05, *Data, Materials, and Software Availability*). In contrast, a subset of 20 metabolites positively correlated with the number of successful learning trials (*SI Appendix*, Fig. S5; Linear mixed effects models corrected with Benjamini–Hochberg method; *P* < 0.05). All but three of these metabolites were more abundant in BeeCom-colonized bees than in at least one other condition (Dataset S2). Most of these metabolites (13 of 20) could be associated with the presence of specific genera, based on their decreased abundance in dropout groups compared to the BeeCom-colonized group (*SI Appendix*, Fig. S6). This shows that several BeeCom members participate in the production of metabolites associated with learning success.

**Fig. 5.**
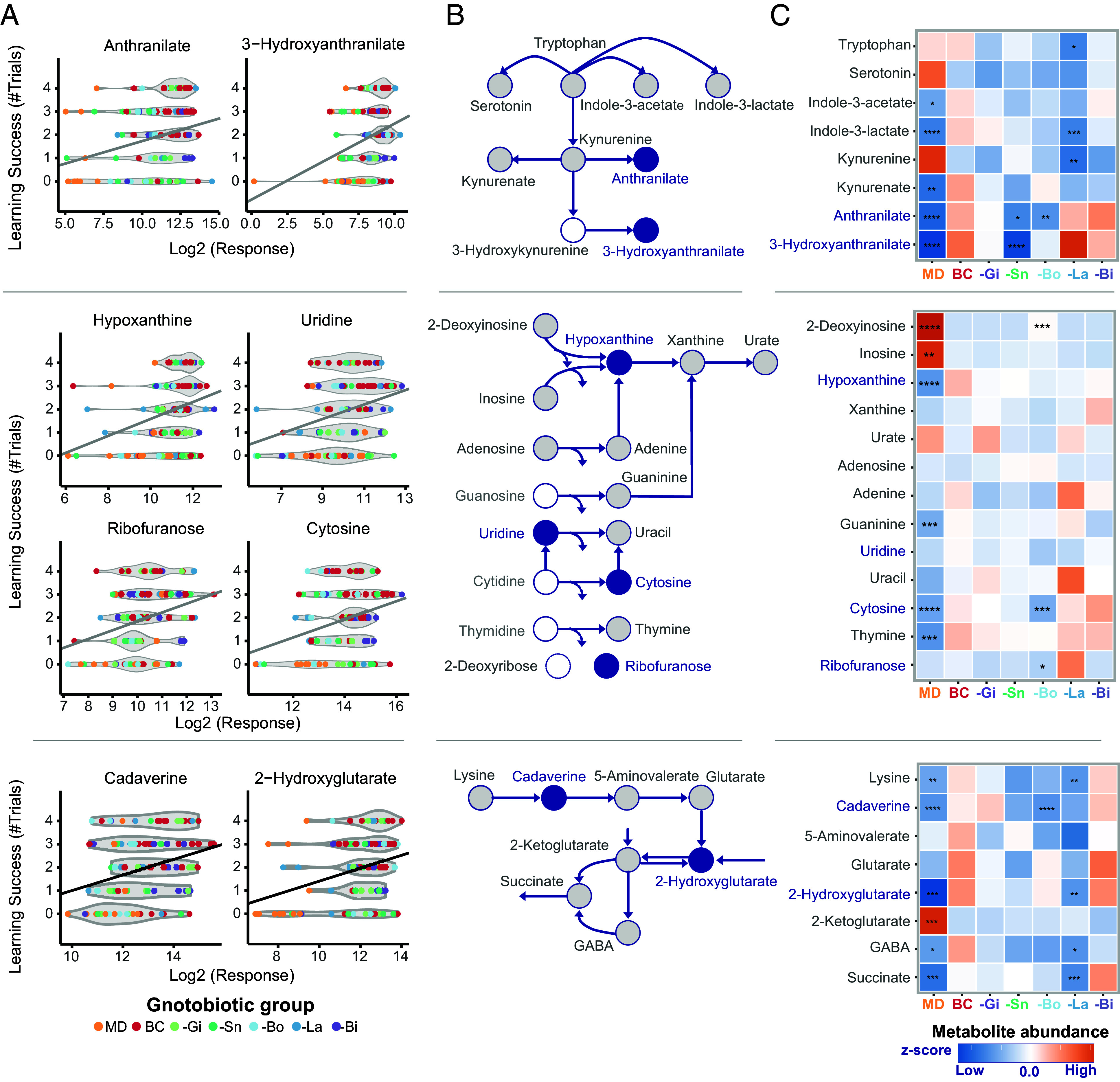
Metabolic pathways modulated by the gut microbiota and affecting learning performance. (*A*) Significant correlations between the learning score and metabolite abundance (Log_2_ normalized) in the gut are highlighted for metabolites resulting from the degradation of tryptophan (*Top*), nucleosides (*Middle*), and lysine (*Bottom*). (*B*) Breakdown of tryptophan, nucleosides, and lysine metabolism are shown with metabolites significantly correlated with learning performance (blue), not significant (gray), and not detected (white). (*C*) Corresponding heatmaps display the median metabolite z-score for the gut colonization group. Significant differences [Wilcoxon rank-sum test with BH correction; *P* < 0.05 (*), *P* < 0.01 (**), *P* < 0.001 (***), and *P* < 0.0001 (****)] compared to the BC condition are indicated.

Many of these metabolites fell within the tryptophan metabolism, nucleoside degradation, or lysine degradation pathways (Fig. 5). Nearly all tryptophan pathway metabolites were sensitive to the microbial composition within the gut. While tryptophan, indole-3-lactate, and kynurenine levels increased with *Lactobacillus* in the gut, the abundances of the kynurenine derivatives anthranilic acid and 3-hydroxyanthranilic acid depended on *Snodgrassella* and *Bombilactobacillus*. These two metabolites were the only tryptophan-derived molecules that were significantly associated with learning success (Fig. 5).

Similarly, nucleotide catabolism liberating the ribose monomer from the corresponding nucleobase appeared to involve multiple bacterial genera, as metabolite abundances in nearly all cases were only significantly different between MD and BeeCom-colonized conditions. The microbes likely catabolized the ribose sugar monomers, leaving behind the nucleobases that were more abundant in colonized than MD guts. However, only hypoxanthine, cytosine, uridine, and ribofuranose were positively associated with enhanced learning performance (Fig. 5). Of these, cytosine and ribofuranose were significantly less abundant when *Bombilactobacillus* was absent.

Finally, lysine catabolites flowing into the TCA cycle were more abundant in BeeCom-colonized than MD bees (Fig. 5). *Lactobacillus* appeared to have the greatest individual impact on these metabolite levels. However, only cadaverine and 2-hydroxyglutarate were positively associated with learning success, and cadaverine’s abundance was only significantly decreased in bees lacking *Bombilactobacillus*.

Taken together, our results highlight multiple metabolic pathways associated with learning performance, all of which were altered by the specific microbial colonization conditions in the gut.

## Discussion

Our study identified a defined synthetic community of cultured native gut bacteria that collectively improves their host’s cognitive abilities. Colonization with individual BeeCom members or with the BeeCom depleted from single members could not reproduce the effect of the community on bees’ learning and memory performance. We identified multiple metabolites connected to tryptophan, lysine, and nucleotide metabolism, whose production by the BeeCom correlates with robust learning performance. Altogether, it suggests that the microbiota-mediated cognitive improvement in bees is an emergent property of the defined community and its metabolic activity.

The use of a dropout experimental approach allowed us to assess the contribution of single community members to the cognitive phenotype promoted by the full defined community. Defined communities have improved our understanding of interspecies interactions among bacterial members as well as transkingdom communication in host–microbe symbioses (19, 20). In *Drosophila melanogaster,* the exchange of metabolites between community members in the gut influenced their host’s olfactory preferences and egg-laying behavior in a different manner compared to individual members (21). Similarly, we did not see any clear effect of individual members on bees’ performance suggesting either that interactions between members trigger the cognitive improvements or that different members have additive effects. Additive and synergistic effects of bacterial strains within a community on host phenotype have also been demonstrated beyond the gut microbiota–brain axis. For instance, pathogen protection is conferred by microbial communities in the gut of mammals (22) and in plants (23), where resistance emerges as a collective property of the community rather than from individual bacterial strains. Our findings align with previous research demonstrating that the natural gut microbiota enhances cognitive function in bees (9, 16). Given the emergent properties described in this study, the relative contribution of our defined synthetic community—compared to the full, native gut microbiota—warrants further investigation. It is likely that other bacteria of the bee gut microbiota participate in maintaining the cognitive performance of their host at physiological levels.

Three independent metabolic pathways involving either *Lactobacillus* or *Gilliamella* strains have been highlighted by previous studies for their positive effects on honey bee and bumblebee cognition (9, 16, 24). In honey bees, *Lactobacillus* colonized alone into bees supplemented with excess tryptophan was shown to increase olfactory long-term memory by transforming tryptophan into indole derivatives that activated aryl hydrocarbon receptors (AhR) on gut epithelial cells, along with a suppression of kynurenine production by the host (9). Our results also home in on microbial tryptophan metabolism as a key factor in host learning performance. While we replicated the metabolic phenotype induced by *Lactobacillus*, we did not observe a corresponding improvement in host learning and short-term memory. Instead, learning performance correlated with changes in the kynurenine pathway of tryptophan metabolism. Specifically, colonization with *Snodgrassella*, or *Bombilactobacillus* increased levels of anthranilic acid and 3-hydroxyanthranillic acid in the gut. While *Snodgrassella* was previously shown to synthesize anthranilic acid from host derived kynurenine (25), differences in kynurenine metabolite levels across treatments highlights the complexity of multistrain interactions and shows that the absence of a single strain from the defined community can cause metabolite shifts beyond those observed between the full community and MD bees. The effect of these nonlinear dynamics on learning performance could be amplified when considering both the metabolic implications of feeding a rich pollen diet instead of supplemented sugar water, as well as the propensity of AhR’s to respond to a broad range of ligands beyond indole derivatives, such as kynurenine and kynurenic acid (26, 27).

Lipid metabolism by the gut microbiota has also been linked to learning performance in bees. Strains of *Gilliamella* metabolize polyunsaturated fatty acids into anandamide, a potent ligand of the endocannabinoid system, while in bumblebees, elevated abundances of *Lactobacillus* within the natural gut community was also shown to promote visual memory performances through glycerophospholipid production (16, 24). Our methods were not designed to detect these specific metabolic phenotypes, making their results incomparable with ours and possibly explaining why no metabolic differences were observed for *Gilliamella* despite its clear effects on bee cognition.

In addition to the above pathways, our results indicate nucleotide and lysine degradation may further contribute to host learning and memory. Strong mechanistic evidence has already linked epigenetic methylation of cytosine in the brain with long-term olfactory memory formation in bees (28–30). It is difficult to perceive a direct link between increased RNA/DNA turnover in the gut and changes in DNA methylation in the brain; however, dietary uridine and cytosine nucleotides have also been shown to enhance membrane phospholipid synthesis and signaling in the brain, with corresponding benefits for memory and learning in mammals (31–33). No such strong evidence exists for effects of lysine catabolism, however it does feed into the TCA cycle and hence could impact levels of the key neurotransmitter GABA. While GABA levels in the gut had no measurable effect on learning performance, the median level was highest in bees colonized with a full community compared to MD bees (*SI Appendix*, Fig. S6).

Neurobiological mechanisms remain somewhat elusive in bees, particularly for AhR activation. There are multiple ways in which the bacterial products of the BeeCom may promote olfactory learning and memory performance. First, they might participate in brain maturation and cognitive development, as observed in humans and mice (34, 35). Honey bees acquire their gut microbiota during perinatal life, concomitantly to structural and functional brain maturation (36, 37). An optimal capacity to memorize odor—food associations was observed between 5 and 8 d after emergence, the age at which we tested bees’ performance in our assay (38, 39). Gut colonization by the BeeCom during the first week of adulthood might have facilitated processes involved in brain maturation which resulted in the reported cognitive improvement. Second, and independently of cognitive development, bacterial products might facilitate the neuronal plasticity triggered by the coincidence detection of the CS and US and required for the formation of memories (40, 41). Adult mice exposed to an antibiotic inducing a dysbiosis in the gut showed reduced learning-related structural and functional plasticity of the neurons involved (7). Further investigations are needed in honey bees to discriminate the effect of the BeeCom on cognitive development from effects on learning processes per se. Sucrose responsiveness did not differ between gnotobiotic groups and was not associated with learning performance in our study, eliminating the possibility that the observed cognitive improvements in bees colonized with the BeeCom are due to the perception of sucrose or appetite.

Our findings on the positive impact of the core gut bacterial community on honey bee cognition provide a basis for further research on the mechanisms, ecology and evolution of the gut microbiota–brain axis. Adopting a systems-level perspective will enable the field to elucidate how complex interactions among community members shape the host phenotype and fitness. The core genera represented in the BeeCom are also present in the gut of other corbiculate bee species, including bumblebees and stingless bees (42). The ecological relevance of this core gut microbiota therefore opens evolutionary questions regarding the selection pressures that apply to corbiculate bees’ cognition and gut bacteria and which have shaped this symbiotic association (43). The value of the BeeCom as a probiotic should also be tested in bees suffering from dysbiosis induced by environmental stressors (44) in an effort to help protect threatened bee populations.

## Materials and Methods

### Generation of Gnotobiotic Bees.

MD honey bees were obtained from 16 colonies of *A. mellifera carnica* maintained on the campus of the University of Lausanne during the spring and summer seasons of 2021, 2022, and 2023. They were generated as described in Kešnerová et al. (45) Briefly, dark-eyed pupae were gently extracted from their wax cell and placed into sterilized plastic boxes in an incubator (35 °C, 75% humidity) for 3 d. A sterility check was performed for each box, by culturing one bee gut homogenate (in 1 mL sterile 1X PBS) on three growth media (*SI Appendix*, Table S1). Any box showing signs of contamination 24 h later was discarded. On day 3 post–pupae extraction, hatched adult bees were randomly assigned to one of the following gnotobiotic groups: MD, colonized by the defined bacterial community *BeeCom_001*, colonized by single community members (monocolonizations; Experiment 1) or by the *BeeCom_001* depleted from single members (dropout communities; Experiment 2). In Experiments 1 and 2, pupae were sourced from 14 and 7 different hives, respectively, with each experimental replicate using pupae from a single hive. Bacterial strains used for the colonization were obtained from existing glycerol stocks stored at −80 °C. Colonization stocks were prepared by culturing each strain under its optimal culturing conditions, restreaking once, washing in 1X PBS, diluting to OD_600_ = 1 and storing in 20% glycerol at −80 °C until further use. Details on bacterial strains and culturing conditions can be found in *SI Appendix*, Table S1. To mimic intragenus diversity, we included several strains per genus, one per species. Colonized bees were obtained by feeding MD bees 5µL of a solution containing the colonization stocks diluted ten times in a 1:1 mixture of 1X PBS and 50% sucrose solution (w/v). The colonization stock used for the MD group consisted of 20% glycerol in 1X PBS. For monocolonization, strains belonging to the same genus (e.g., Gilliamella) were mixed in equal proportions before being diluted in the PBS/sucrose solution. The *BeeCom_001* and drop-out communities contained all strains in equal proportions.

Colonized bees were housed in cages of 10 to 19 individuals depending on the mortality encountered during the generation of MD bees. For a given replicate, all treatment groups contained the same number of individuals. The monocolonization and dropout experiments were replicated 14 and 9 times, respectively. Bees had unlimited access to 50% sucrose solution (w/v) and to sterilized pollen. Cages were kept at 30 °C with 70% humidity for 7 d. On the last evening, bees were transferred to clean cages for a 12-h overnight fasting.

### Sucrose Responsiveness assay.

Sucrose responsiveness was assessed as described in Scheiner et al. (46). On the eighth day postinoculation, bees were anesthetized on ice and harnessed in 3D-printed tubes allowing movements of the antennae and mouthparts only. After 2 h of rest in darkness, bees were presented with an ascending concentration series of sucrose (i.e., 0.1, 0.3, 1.0, 3.0, 10, 30% w/v) applied to both antennae simultaneously. Each sucrose stimulation was preceded by a stimulation with water. The presence or absence of PER to each stimulation was recorded. The interval between two stimulations of the antennae was 3 min. Responses to water reflect the thirst of the tested individuals, as well as the phenomenon of sensitization occurring when individuals are repeatedly stimulated. Bees responding to water were therefore excluded from the analysis of sucrose responsiveness. The sucrose response score was calculated for each individual as the number of sucrose concentrations that elicited proboscis extension (range 0 to 6). For the olfactory conditioning assay, a subset of bees was randomly selected from those who responded to the highest sucrose concentration, which served as the unconditioned stimulus in the conditioning assay. This selection was necessary due to time constraints imposed by the intertrial interval and the trial duration which limit the number of bees that can be conditioned.

### Olfactory Conditioning.

The protocol for the olfactory conditioning of the PER was adapted from Matsumoto et al. (47) and started after 1 h of rest for experiment 1. The experimenter was blinded with respect to the gnotobiotic group. Bees were trained to discriminate an odor associated with a sucrose reward (conditioned stimulus; CS+) from an unrewarded odor (CS−). The odors heptanal and 1-nonanol (Sigma-Aldrich) were used alternately as CS+ or CS− between each replicate. They were chosen based on their medium level of perceptual similarity (48). The conditioning assay included in five trials of 40 s for each odor, presented in a pseudorandomized order. For each trial, bees were placed on the conditioning set-up, in front of a syringe containing a filter paper soaked with 5 µL of pure odorant. After 12 s of familiarization with the context, a constant airflow was sent through the syringe, thereby delivering the odor to the bee for 4 s. A toothpick soaked in 50% sucrose solution (w/v) was presented to the bees’ antennae 3 s after the onset of CS+ delivery. Bees extending their proboscis were then allowed to drink the sucrose solution for 3 s. The intertrial interval was 10 min. The presence or absence of PER during the odor presentations was noted as 1 or 0, respectively. Bees responding to the first presentation of the CS+ and bees showing no PER to the sucrose stimulations were discarded from the analysis (<5% of the bees). Short-term memory was assessed by presenting both CS in a randomized order 15 min after the end of the conditioning experiment. The guts of the tested bees were collected immediately after the memory test and stored at −80 °C for further analyses.

### DNA Extraction From Honeybee Gut Tissue.

One gut per gnotobiotic group and experimental replicate was processed for DNA extractions and qPCR analyses. All guts of *BeeCom*-colonized bees were processed in the case of Experiment 2. The protocol for DNA extractions was adapted from Kešnerová et al. (45). Samples were homogenized with 750µL of deionized water, zirconia beads (0.1 mm dia. Zirconia/Silica beads; Carl Roth), and glass beads in a Fast- Prep24 5G homogenizer (MP Biomedicals) at 6 m/s for 45 s. Half of the gut homogenate was removed and stored at −80 °C as a backup. Samples (still containing the beads) were homogenized again with 375 µL of CTAB lysis buffer (0.2 M Tris-HCl, pH 8; 2.8 M NaCl; 0.04 M EDTA, pH 8; 4% CTAB, w/v, dissolved at 56 °C; 2 µL β-mercaptoethanol; 20 µL proteinase K [20 mg/mL]) and incubated at 56 °C for 1 h. Homogenates were mixed with 750 µL phenol:chloroform:isoamyl alcohol (Fischer Bioreagents, pH 8), centrifuged at 16,000×*g* for 10 min at room temperature. The upper aqueous phase (500 μL) was transferred to a tube containing the same volume of chloroform, mixed, and centrifuged again at 16,000×*g* for 10 min at room temperature. The upper aqueous phase (500 μL) was mixed with 900 μL of precooled 100% ethanol and incubated overnight at −20 °C for precipitation of nucleic acids. After a centrifugation at 16,000×*g* at 4 °C for 30 min, the pellets were washed with 900 μL of 70% ethanol, left to dry at room temperature and resuspended in 50 μL of nuclease-free water (Invitrogen) by shaking in a thermomixer (64 °C, 400 rpm, 10 min). The purification of nucleic acids was performed using CleanNGS magnetic beads (CNGS-0005) and the Opentron OT-2 pipetting robot. Purified DNA extracts were stored at −20 °C until further use.

### Quantification of Bacterial Loads in the Gut.

All qPCRs were carried out in a 96-well plate on a QuantStudio™ 5 (Applied Biosystems). The thermal cycling conditions were as follows: denaturation stage at 50 °C for 2 min followed by 95 °C for 2 min, 40 amplification cycles at 95 °C for 15 s, and 60 °C for 1 min. Each reaction was performed in triplicate in a total volume of 10 µL (0.2 µM of each forward and reverse primer; 1× SYBR® Select Master Mix, Applied Biosystems; 1 µL DNA). Each DNA sample was screened with two different sets of primers targeting either the actin gene of *A. mellifera*, or the universal 16S rRNA region. Samples from MD bees, bees colonized with the BeeCom_001 and bees colonized with the dropout communities were screened with the five sets of species-specific primers. Samples from monocolonized bees were screened with the set of species-specific primers corresponding to the genus present in the inoculum. Information about primers and absolute quantification method can be found in Kešnerová et al. (45).

### GC–MS Analysis of Metabolites.

Metabolomic analyses were performed on the same gut samples as the ones collected for qPCR in Experiment 2. Homogenized bee gut extracts were centrifuged at 4,000×*g*^−1^ for 20 min at 4 °C and frozen at −70 °C until extraction. Extraction was performed after addition of internal standards (ISTDs) for 2 h with a 1:1 methanol:acetonitrile solvent at −20 °C. Samples were again centrifuged, and the supernatant vacuum evaporated at ambient temperature. Metabolites were derivatized first with 20 mg/mL of methoxyamine hydrochloride in pyridine at 33 °C for 1.5 h, followed by silylation with MSTFA for 2 h at 45 °C. Samples were kept at 15 °C for up to 24 h postderivatization and analyzed on an Agilent 8890-5977B GC-MSD equipped with a Pal3 autosampler that injected 1 μL onto a VF-5MS (30 m × 0.25 mm × 0.25 mm) column. The samples were injected with a split ratio of 15:1, helium flow rate of 1 mL/min and inlet temperature of 280 °C. The temperature was held for 2 min at 125 °C, raised at 3 °C/min to 150 °C, 5 °C/min to 225 °C, 15 °C/min to 310 °C and held for 3.3 min. The MSD was run in SIM/Scan mode with 3 ions selected for each targeted metabolite and a scan from 50 to 600 Da. Data analysis of untargeted features was performed with MZmine 4.3 and the NIST 2017 MS library (49). Top library matches are reported for features with a minimum cosine similarity of 0.7. Analyte abundances of known compounds with pure analytical standards were calculated using the MassHunter Quantitative Analysis software (Agilent). All reagents and standards were purchased from Sigma Aldrich.

### Statistical Analyses.

All statistical analyses were performed with R Studio 2022.02.0 (50) and significance was set at *P* < 0.05. The presence or absence of PER to the CS during the olfactory conditioning and memory test was recorded as a binomial variable. Generalized linear mixed models with a binomial error structure—logit-link function—[“glmer” function from the lme4 package (51)] were used to assess the predictive value of the trials, gnotobiotic group and odor on responses to the CS. The MD and BeeCom groups were used as reference levels for the models. Replicate and individual identity were set as nested random factors. Pairwise post hoc analyses corrected with the FDR method were run for each trial whenever a statistical difference was detected in the models. The first successful trial, i.e., the first trial in which individual bees respond to the rewarded odor, was compared between gnotobiotic groups using a Dunn test with FDR correction. The Kruskal–Wallis test, followed by pairwise Wilcoxon Rank Sum tests corrected with the FDR method were used to compare mortality, sucrose consumption, carcass weight, and sucrose response scores between the different gut conditions.

Metabolite abundances were processed with custom R scripts. Raw metabolite abundances were normalized to the internal standards. Low-quality samples and samples with an ISTD response < or > two SD from the batch mean were removed from the datasets. To assess differences in gut metabolic profiles across treatment groups, metabolite concentration data were standardized using z-score scaling and a permutational multivariate ANOVA [PERMANOVA; “adonis2” function from the vegan package (52)] using Euclidean distance and 999 permutations was performed. When a significant effect was detected, pairwise comparisons between treatment groups were performed [“pairwise.adonis”, pairwiseAdonis (53)]. *P*-values were adjusted for multiple comparisons using the Benjamini–Hochberg (BH) method. To quantify changes in metabolite abundance between treatment groups, we calculated the log^2^ fold-change values for each metabolite. Statistical significance of differences in normalized metabolite levels between groups was assessed using a Wilcoxon rank-sum test and corrected for multiple testing using the Benjamini–Hochberg method.

The number of successful learning trials was calculated for each individual as the difference between the number of responses to the CS+ and the number of responses to the CS− across the five learning trials. The correlation between the number of successful learning trials and the log2 normalized metabolite abundance was calculated with a mixed effects model with hive as a random effect using the lme4 package. The *P*-values of the best fit slopes were adjusted for multiple comparisons using the BH method. A random forest analysis implemented with the caret package attempted to link log2 normalized metabolite abundances to successful performance on the pass/fail memory performance assay, with 80% of the samples randomly assigned to the training set and 20% assigned to the test set.

## Supplementary Material

Appendix 01 (PDF)

Dataset S01 (XLSX)

Dataset S02 (XLSX)

## Data Availability

The complete set of data used for the statistical analyses, computational code used to analyze data, and generate figures have been deposited on Zenodo at https://doi.org/10.5281/zenodo.18929127. The metabolomics data have been deposited to MetaboLights (54) repository with the study identifier MTBLS13943.
