# Supplementary material for: A defined community of core gut microbiota members promotes cognitive performance in honey bees
Source: Proc Natl Acad Sci U S A. 2026 May 20;123(21):e2608600123. doi: 10.1073/pnas.2608600123 (PMC13214017; doi:10.1073/pnas.2608600123)
Supplement: Supplementary file 1 — Appendix 01 (PDF) [file pnas.2608600123.sapp.pdf]

Supplementary Table S1. Bacterial strains used in this study and their culturing conditions. The proportion of each strain relative to other strains present in the inoculum of the different gut treatments is provided under brackets. The defined community BeeCom_001 (BC) contained all 11 strains.

Bacterial species	Strain name	Gut treatments	Culturing condition
Gilliamella apicola	ESL0178	BC (1:11), Gi (1:3), -Gi (1:8)	BHIA, 35°C, 5% CO_2
Gilliamella apis	ESL0169	BC (1:11), Gi (1:3), -Gi (1:8)	BHIA, 35°C, 5% CO_2
Gilliamella sp.	ESL0177	BC (1:11), Gi (1:3), -Gi (1:8)	BHIA, 35°C, 5% CO_2
Snodgrassella alvi	ESL0145 (wkB2)	BC (1:11), Sn (1:1), -Sn (1:10)	TSA, 35°C, 5% CO2
Bombilactobacillus mellis	ESL0094 (Hon2N)	BC (1:11), Bo (1:1), -Bo (1:10)	MRSA, 37°C, anaerobic
Lactobacillus apis	ESL0185 (Hma11)	BC (1:11), La (1:4), -La (1:7)	MRSA, 37°C, anaerobic
Lactobacillus helsingborgensis	ESL0183 (Bma5)	BC (1:11), La (1:4), -La (1:7)	MRSA, 37°C, anaerobic
Lactobacillus melliventris	ESL0184 (Hma8)	BC (:11), La (1:4), -La (1:7)	MRSA, 37°C, anaerobic
Lactobacillus kullabergensis	ESL0186 (Biut2)	BC (1:11), La (1:4), -La (1:7)	MRSA, 37°C, anaerobic
Bifidobacterium asteroides	ESL0170	BC (1:11), Bi (1:2), -Bi (1:9)	MRSA, 37°C, anaerobic
Bifidobacterium asteroides	ESL0197	BC (1:11), Bi (1:2), -Bi (1:9)	MRSA, 37°C, anaerobic

Dataset S1: Metabolite fold-change and corrected p-value compared to BeeCom colonization

(.xls file)

Dataset S2: Metabolite z-score per condition

(.xls file)

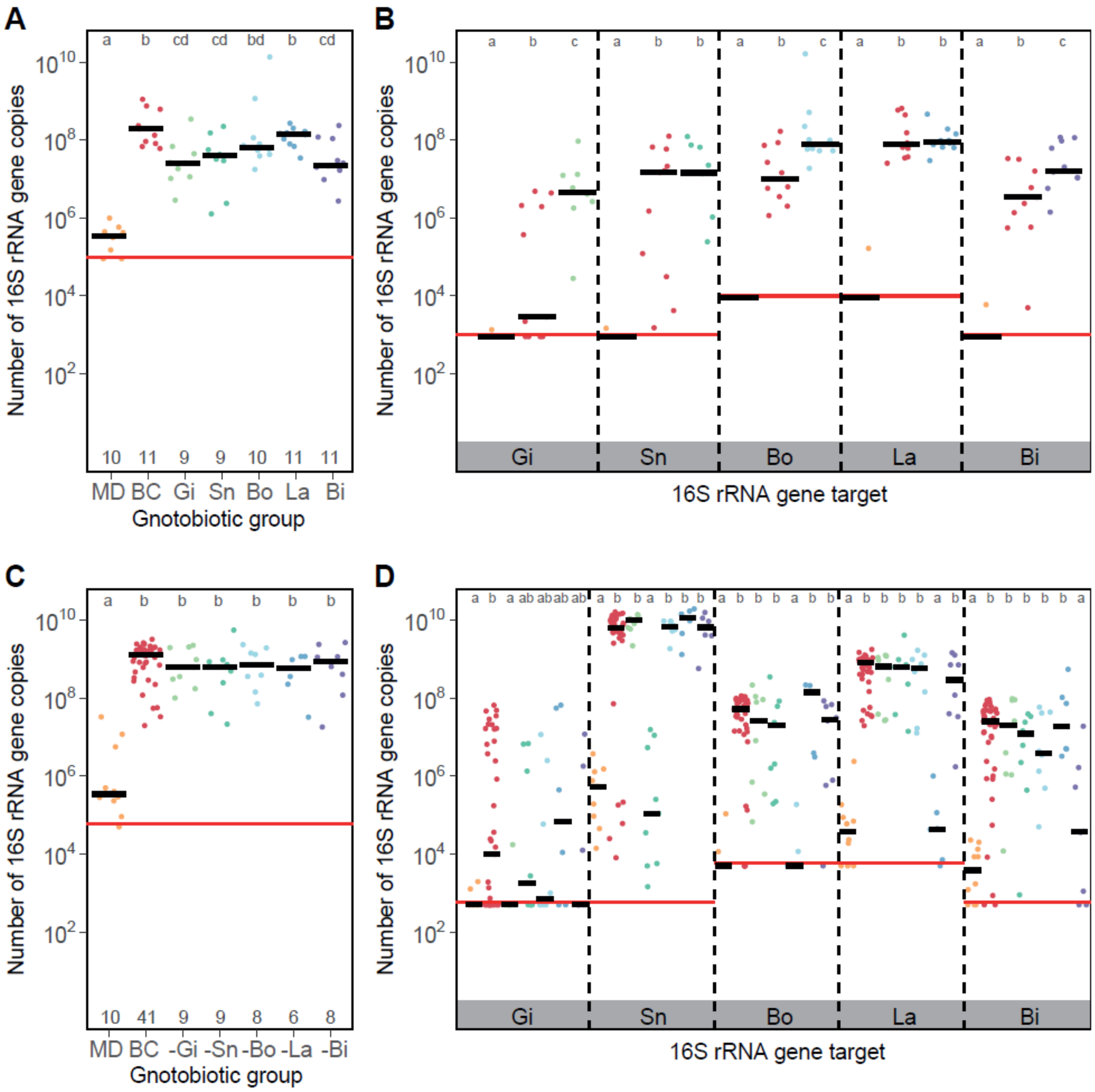

Supplementary Figure S1. Bacterial loads in the gut of microbiota-deprived bees (MD), bees colonized with the defined community BeeCom (BC) composed of the five core members of the bee gut microbiota, and bees mono-colonized with **(A-B)** or depleted from **(C-D)** one of the BeeCom members: *Gilliamella* (Gi), *Snodgrassella* (Sn), *Bombilactobacillus* (Bo), *Lactobacillus* (La), or *Bifidobacterium* (Bi). Gene copy number was measured by qPCR using universal 16S rRNA gene primers detecting all bacteria **(A-C)** or using species-specific primers **(B-D)**. The red lines represent the limit of detection of each primer pair under which bacterial loads were too low to be accurately measured. Different letters at the top indicate significant differences between gnotobiotic groups for each primer pair (Wilcoxon Rank Sum test, false-discovery rate correction).

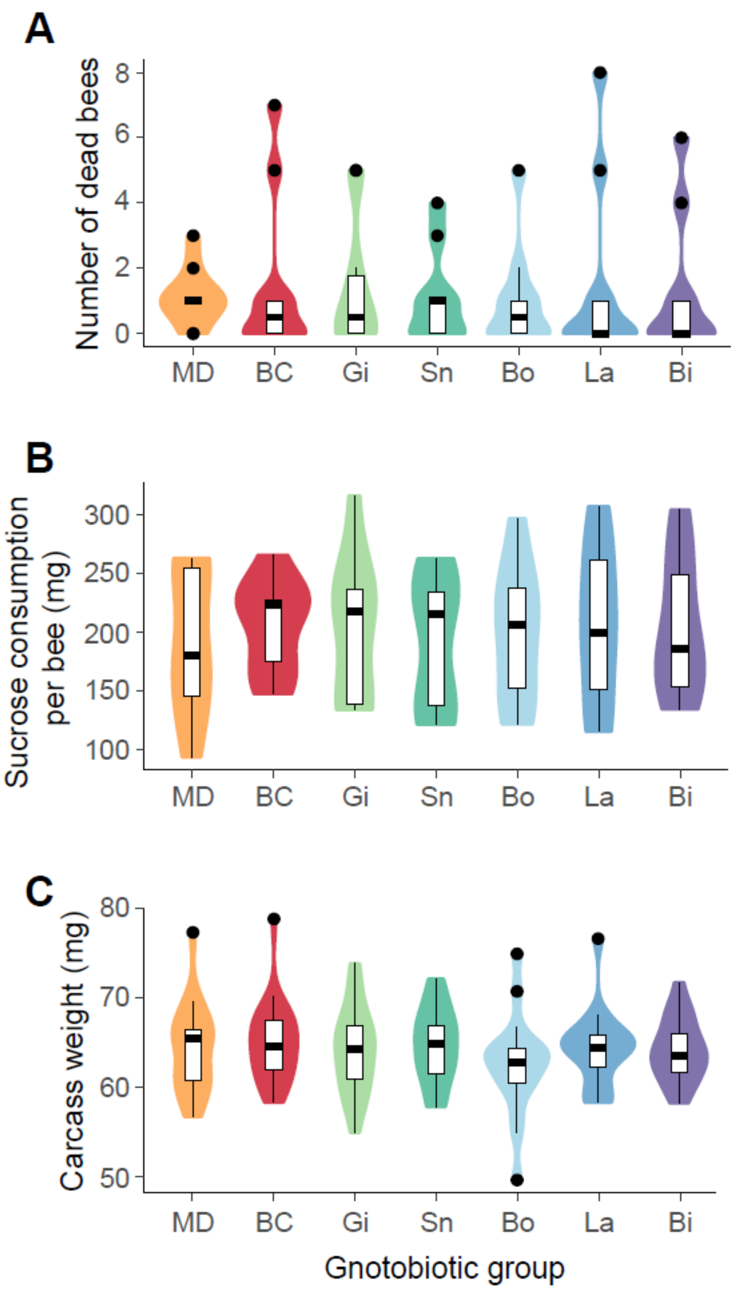

Supplementary Figure S2. Impact of the gut condition on bees' physiology. (A) The number of dead bees and **(B)** the sucrose consumption in the rearing cages were recorded for 7 days during Experiment 1 (n = 10 cages per gut condition). The sucrose consumption per cage was divided by the number of bees in the cage each day. **(C)** The carcass weight of bees was measured following gut dissections. The gut conditions were microbiota-deprived (MD, n = 21), colonized with the defined community BeeCom (BC, n = 29), and mono-colonized with one BeeCom member: *Gilliamella* (Gi, n = 23), *Snodgrassella* (Sn, n = 21), *Bombilactobacillus* (Bo, n = 19), *Lactobacillus* (La, n = 20), or *Bifidobacterium* (Bi, n = 22). Kruskall-Wallis tests were non-significant (Mortality: χ^2 = 1.43, df = 6, p = 0.96; Consumption: χ^2 = 0.6, df = 6, p = 1; Carcass weight: χ^2 = 3.37, df = 6, p = 0.76).

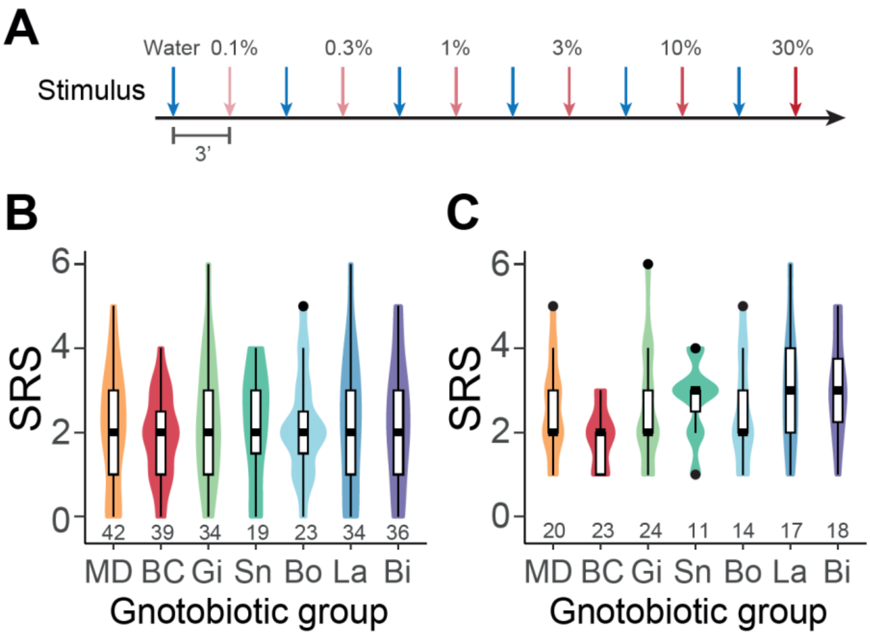

Supplementary Figure S3. Impact of gut bacteria on sucrose responsiveness. (A) Sucrose responsiveness was measured by recording the presence or absence of proboscis extension response to an increasing concentration series of sucrose (*red arrows*) interspersed with water stimulations. **(B)** Sucrose response scores (SRS) were calculated as the sum of sucrose concentrations individual bees responded to. The SRS did not differ significantly between microbiota-depleted (MD) bees, bees colonized with the defined community BeeCom (BC), and bees mono-colonized with *Gilliamella* (Gi), *Snodgrassella* (Sn), *Bombilactobacillus* (Bo), *Lactobacillus* (La), or *Bifidobacterium* (Bi). Kruskal-Wallis test; $\chi 2 = 1.90$, df = 6, $p = 0.93$. **(C)** In the subset of bees randomly selected for the conditioning assay, BC-colonized bees showed a significantly lower SRS than Sn-, La- and Bi-colonized bees (Wilcoxon test corrected with FDR procedure; $p < 0.05$ for these three comparisons). Sample sizes are low in this subset and the differences observed are likely false-positives as the selection process was fully random. Pairwise wilcoxon tests corrected with FDR procedure; $p < 0.05$ (*). Sample sizes are provided below the violin plots

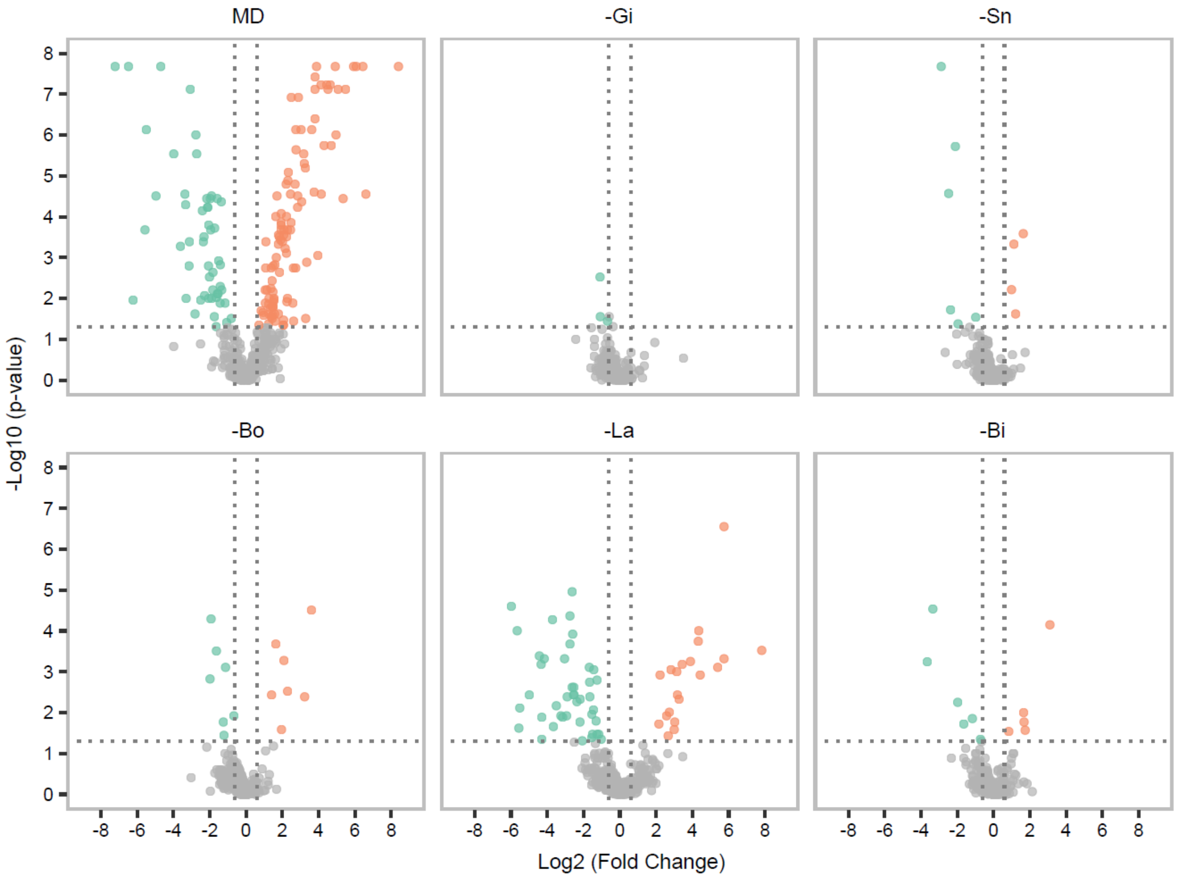

Supplementary Figure S4: Gut metabolite changes between BeeCom-colonized bees and other gnotobiotic groups. Fold-change in metabolite abundance showing metabolites upregulated (red) or downregulated (green) in BeeCom-colonized bees compared to microbiota-deprived (MD) bees and bees colonized with the BeeCom lacking *Gilliamella* (-Gi), *Snodgrassella* (-Sn), *Bombilactobacillus* (-Bo), *Lactobacillus* (-La) or *Bifidobacterium* (-Bi).

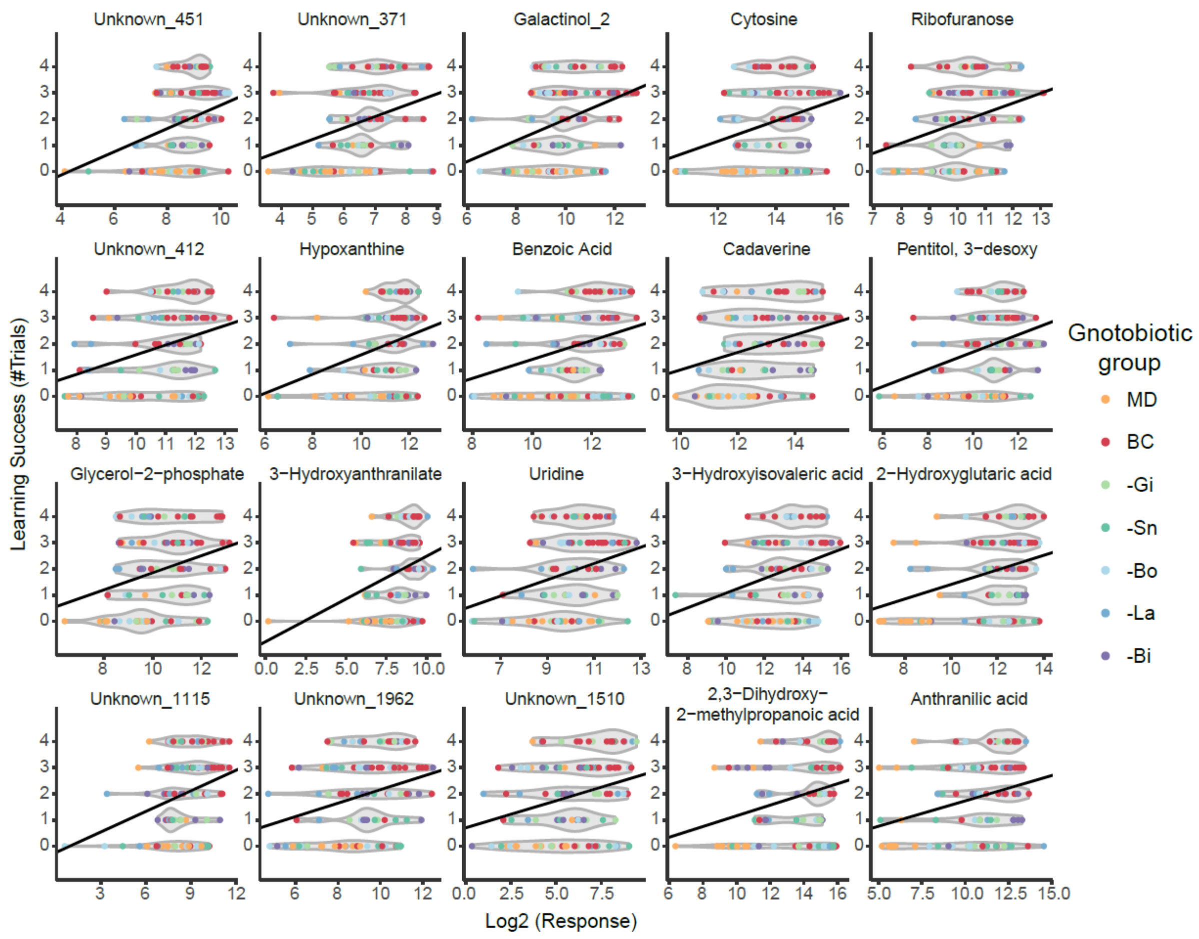

Supplementary Figure S5: Metabolite abundance effects on learning success. Metabolite abundances (Log_2 normalized) are plotted against the number of times a bee correctly responded to the olfactory learning test. Points are colored by gnotobiotic group. The calculated intercept and slope of the mixed linear effects model are plotted as a black line. Only significant metabolites after multiple testing correction are plotted.

Supplementary Figure S6: Metabolite abundances across colonization conditions. Relative metabolic feature abundances (z-score) are plotted for each gnotobiotic group. Significance is shown for each group compared to the BeeCom-colonized bees (Wilcoxon rank sum test with BH correction; $p<0.05$ (*), $p<0.01$ (**), $p<0.001$ (***)).

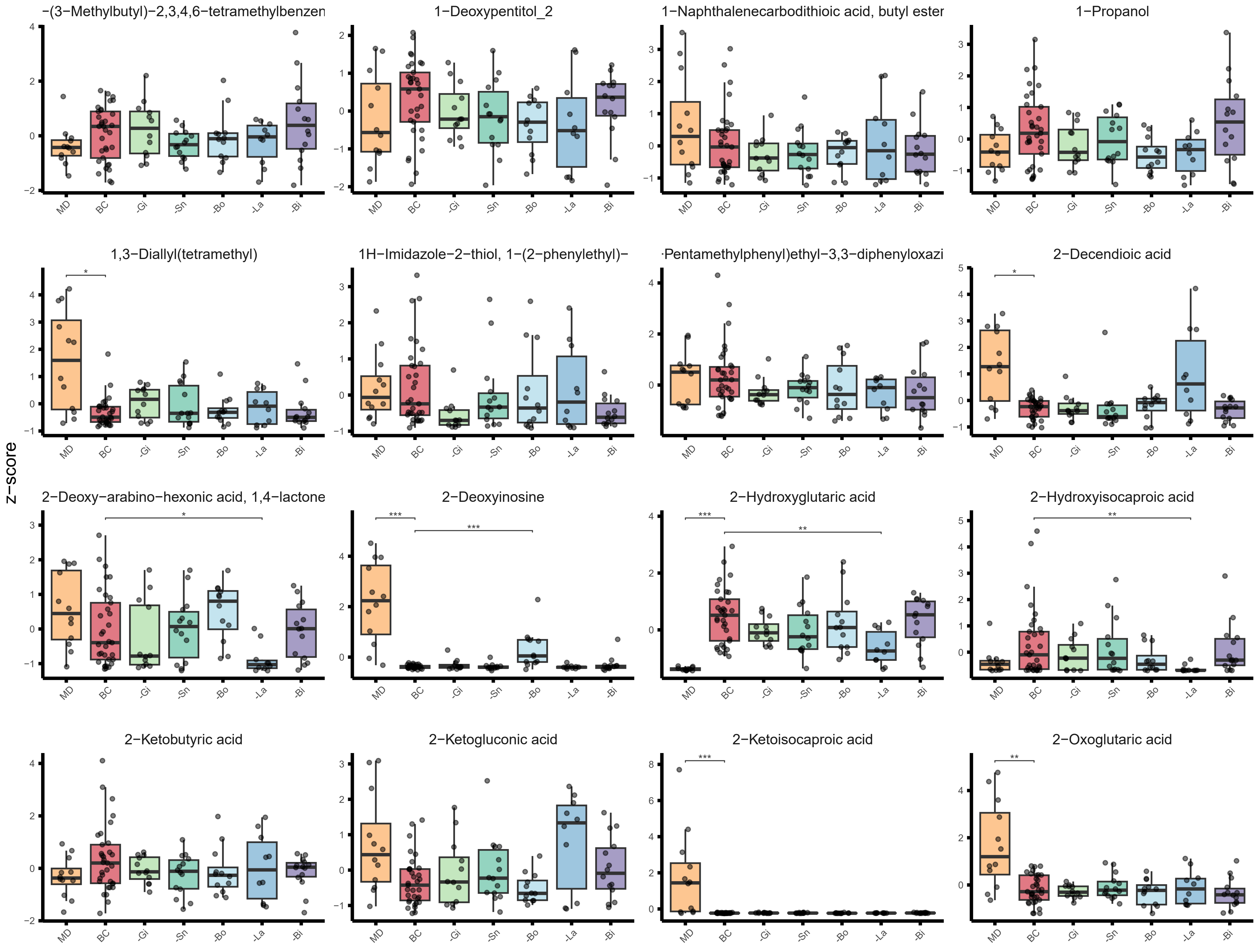

z−score
−(3−Methylbutyl)−2,3,4,6−tetramethylbenzen
1−Deoxypentitol_2
1−Naphthalenecarbodithioic acid, butyl ester
1−Propanol
1,3−Diallyl(tetramethyl)
1H−Imidazole−2−thiol, 1−(2−phenylethyl)−
·Pentamethylphenyl)ethyl−3,3−diphenyloxazi
2−Decendioic acid
2−Deoxy−arabino−hexonic acid, 1,4−lactone
2−Deoxyinosine
2−Hydroxyglutaric acid
2−Hydroxyisocaproic acid
2−Ketobutyric acid
2−Ketogluconic acid
2−Ketoisocaproic acid
2−Oxoglutaric acid
MD
BC
-Gi
-Sn
-Bo
-La
-Bi

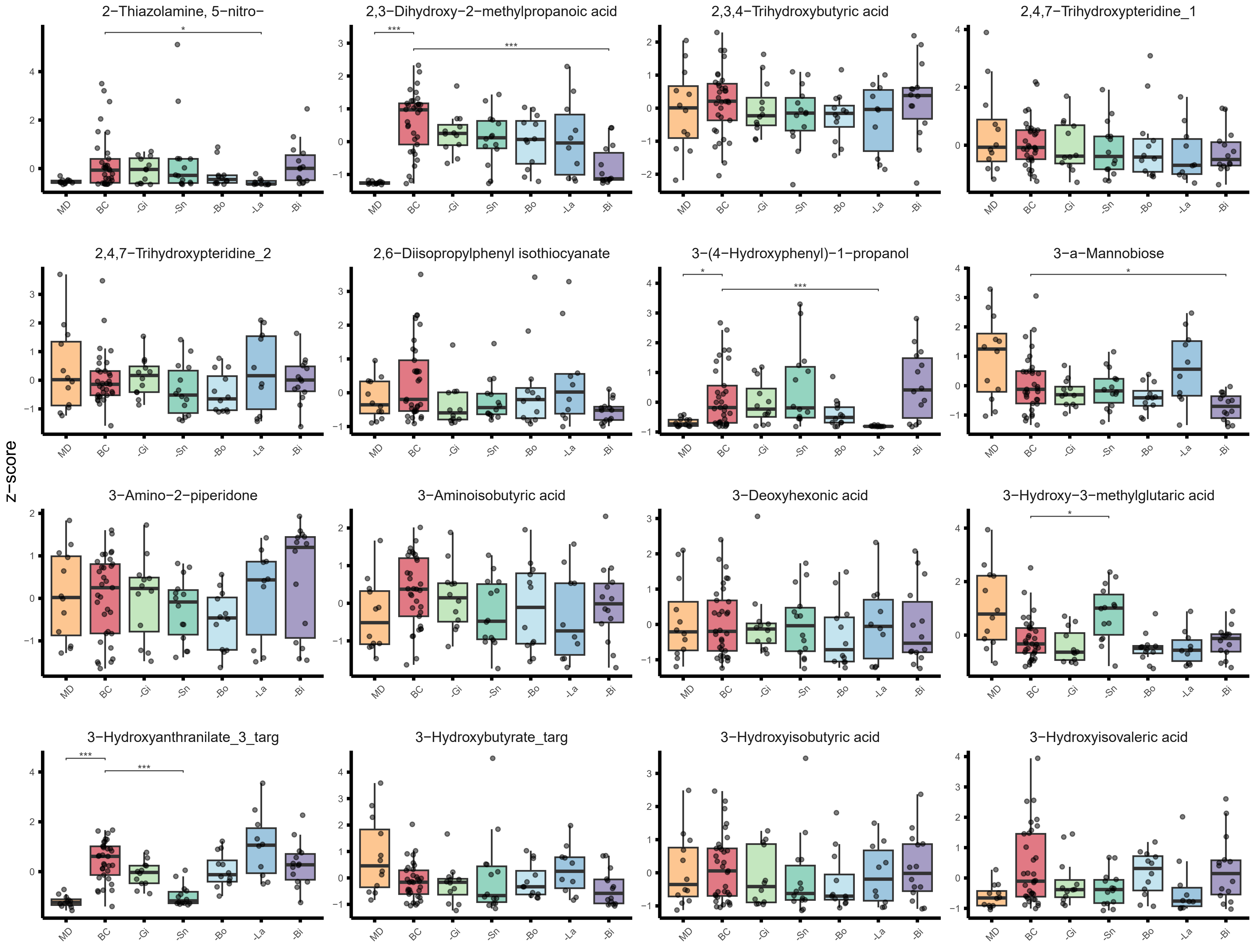

2−Thiazolamine, 5−nitro−
2,3−Dihydroxy−2−methylpropanoic acid
2,3,4−Trihydroxybutyric acid
2,4,7−Trihydroxypteridine_1
2,4,7−Trihydroxypteridine_2
2,6−Diisopropylphenyl isothiocyanate
3−(4−Hydroxyphenyl)−1−propanol
3−a−Mannobiose
3−Amino−2−piperidone
3−Aminoisobutyric acid
3−Deoxyhexonic acid
3−Hydroxy−3−methylglutaric acid
3−Hydroxyanthranilate_3_targ
3−Hydroxybutyrate_targ
3−Hydroxyisobutyric acid
3−Hydroxyisovaleric acid
z−score
MD
BC
-Gi
-Sn
-Bo
-La
-Bi

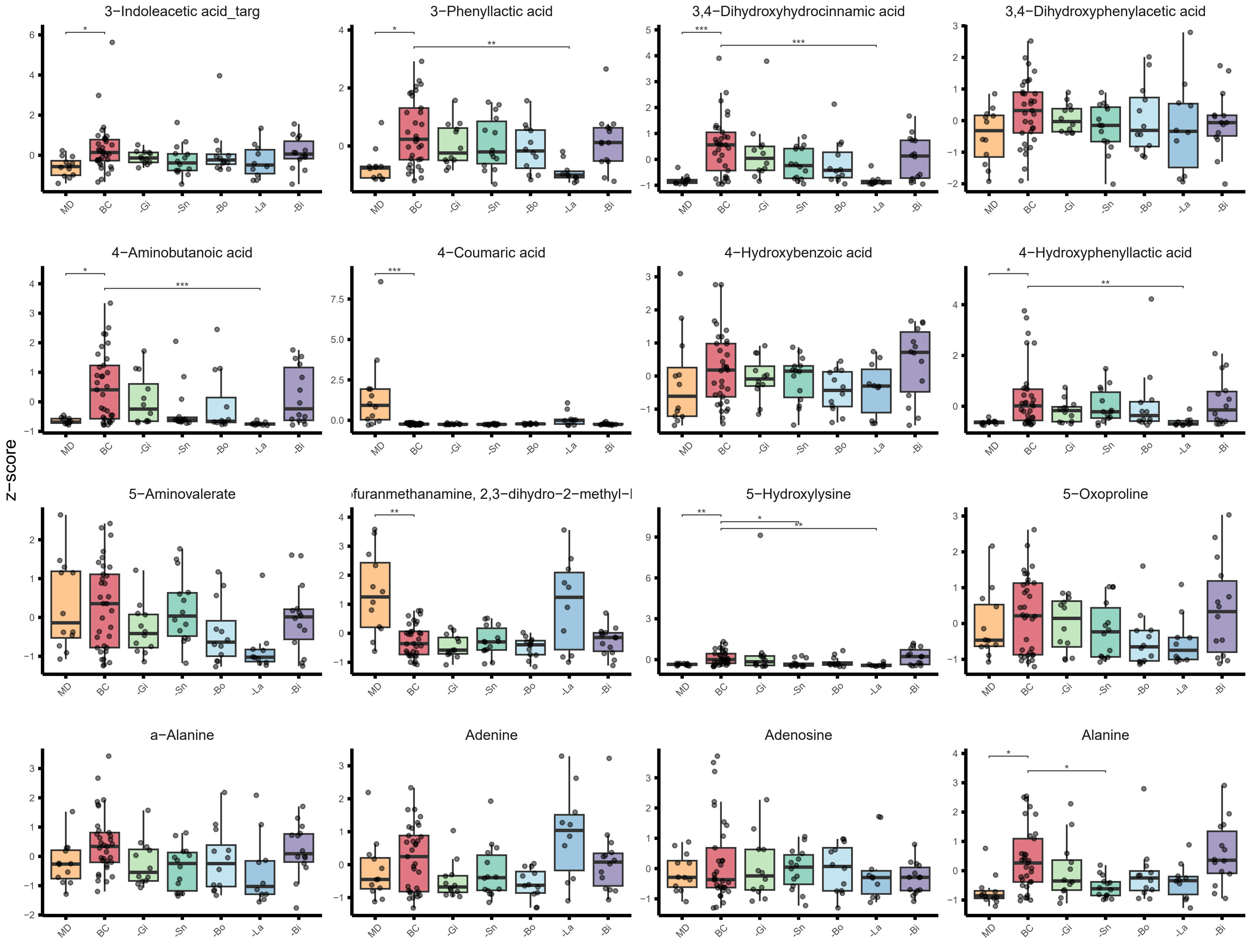
3−Indoleacetic acid_targ
3−Phenyllactic acid
3,4−Dihydroxyhydrocinnamic acid
3,4−Dihydroxyphenylacetic acid
4−Aminobutanoic acid
4−Coumaric acid
4−Hydroxybenzoic acid
4−Hydroxyphenyllactic acid
5−Aminovalerate
furanmethanamine, 2,3−dihydro−2−methyl−
5−Hydroxylysine
5−Oxoproline
a−Alanine
Adenine
Adenosine
Alanine
z−score
MD
BC
-Gi
-Sn
-Bo
-La
-Bi

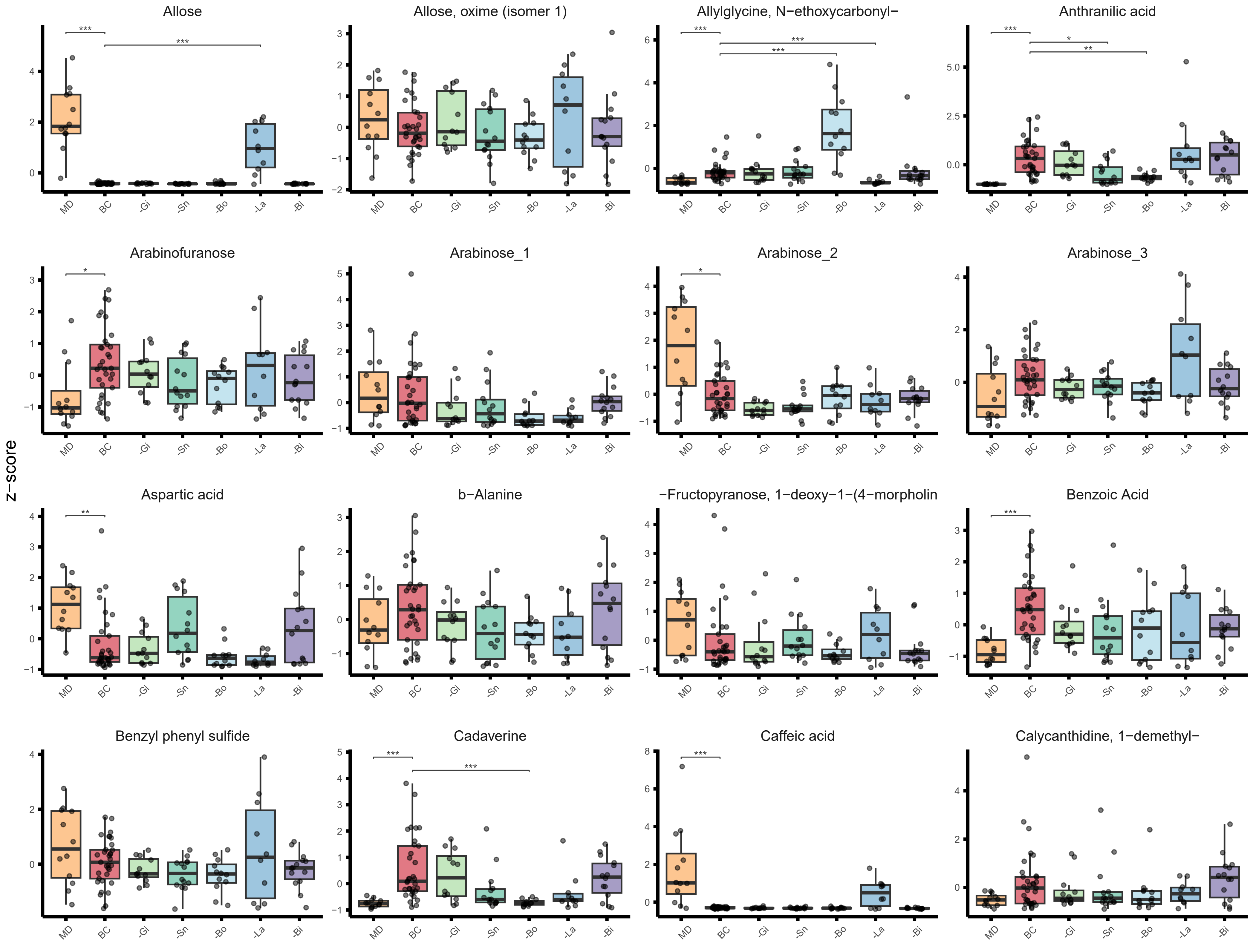
Allose
Allose, oxime (isomer 1)
Allylglycine, N−ethoxycarbonyl−
Anthranilic acid
Arabinofuranose
Arabinose_1
Arabinose_2
Arabinose_3
Aspartic acid
b−Alanine
−Fructopyranose, 1−deoxy−1−(4−morpholin
Benzoic Acid
Benzyl phenyl sulfide
Cadaverine
Caffeic acid
Calycanthidine, 1−demethyl−
z−score
MD
BC
-Gi
-Sn
-Bo
-La
-Bi

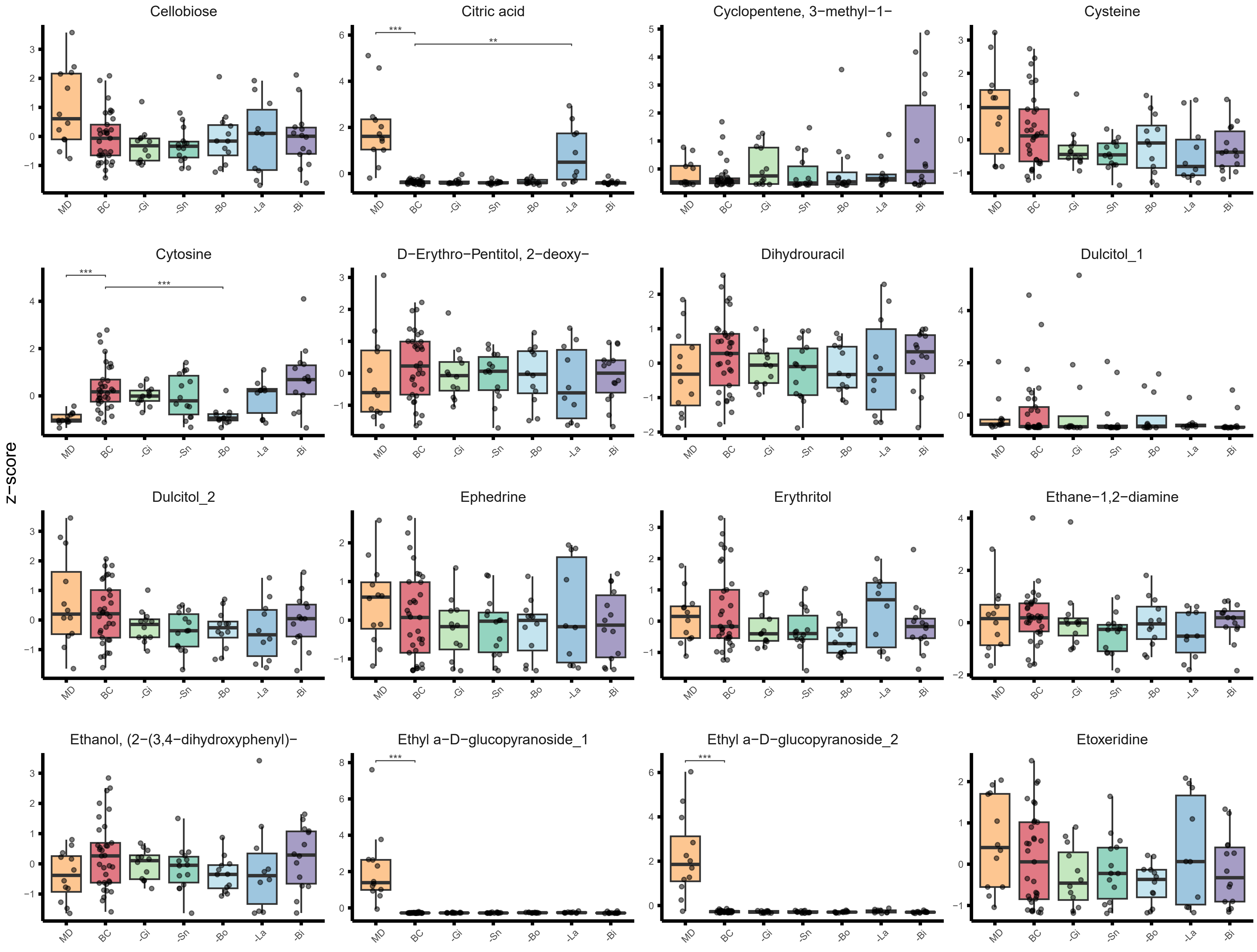

z−score
Cellobiose
Citric acid
Cyclopentene, 3−methyl−1−
Cysteine
Cytosine
D−Erythro−Pentitol, 2−deoxy−
Dihydrouracil
Dulcitol_1
Dulcitol_2
Ephedrine
Erythritol
Ethane−1,2−diamine
Ethanol, (2−(3,4−dihydroxyphenyl)−
Ethyl a−D−glucopyranoside_1
Ethyl a−D−glucopyranoside_2
Etoxeridine
MD
BC
-Gi
-Sn
-Bo
-La
-Bi
***
**

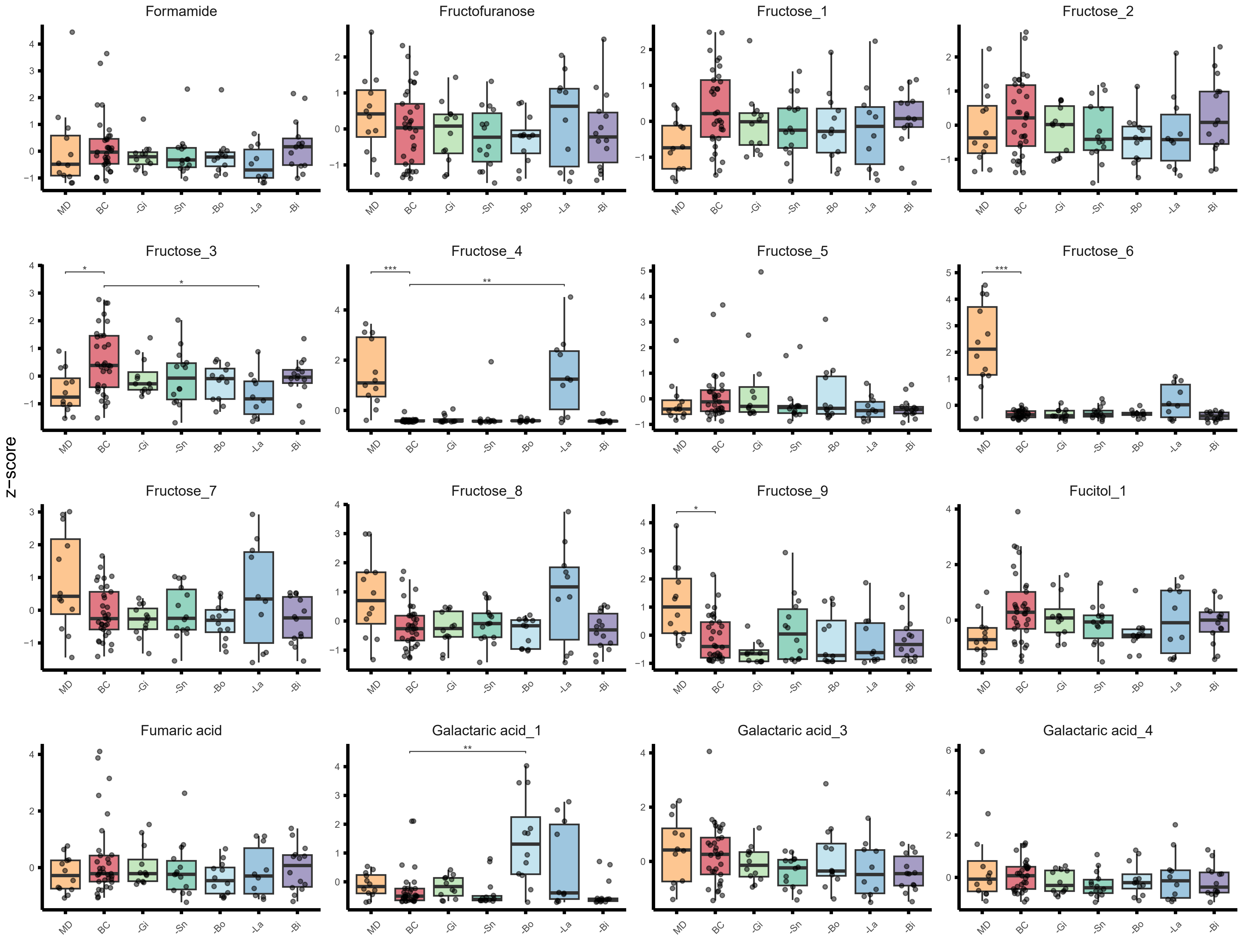

z−score
Formamide
Fructofuranose
Fructose_1
Fructose_2
Fructose_3
Fructose_4
Fructose_5
Fructose_6
Fructose_7
Fructose_8
Fructose_9
Fucitol_1
Fumaric acid
Galactaric acid_1
Galactaric acid_3
Galactaric acid_4
MD
BC
-Gi
-Sn
-Bo
-La
-Bi

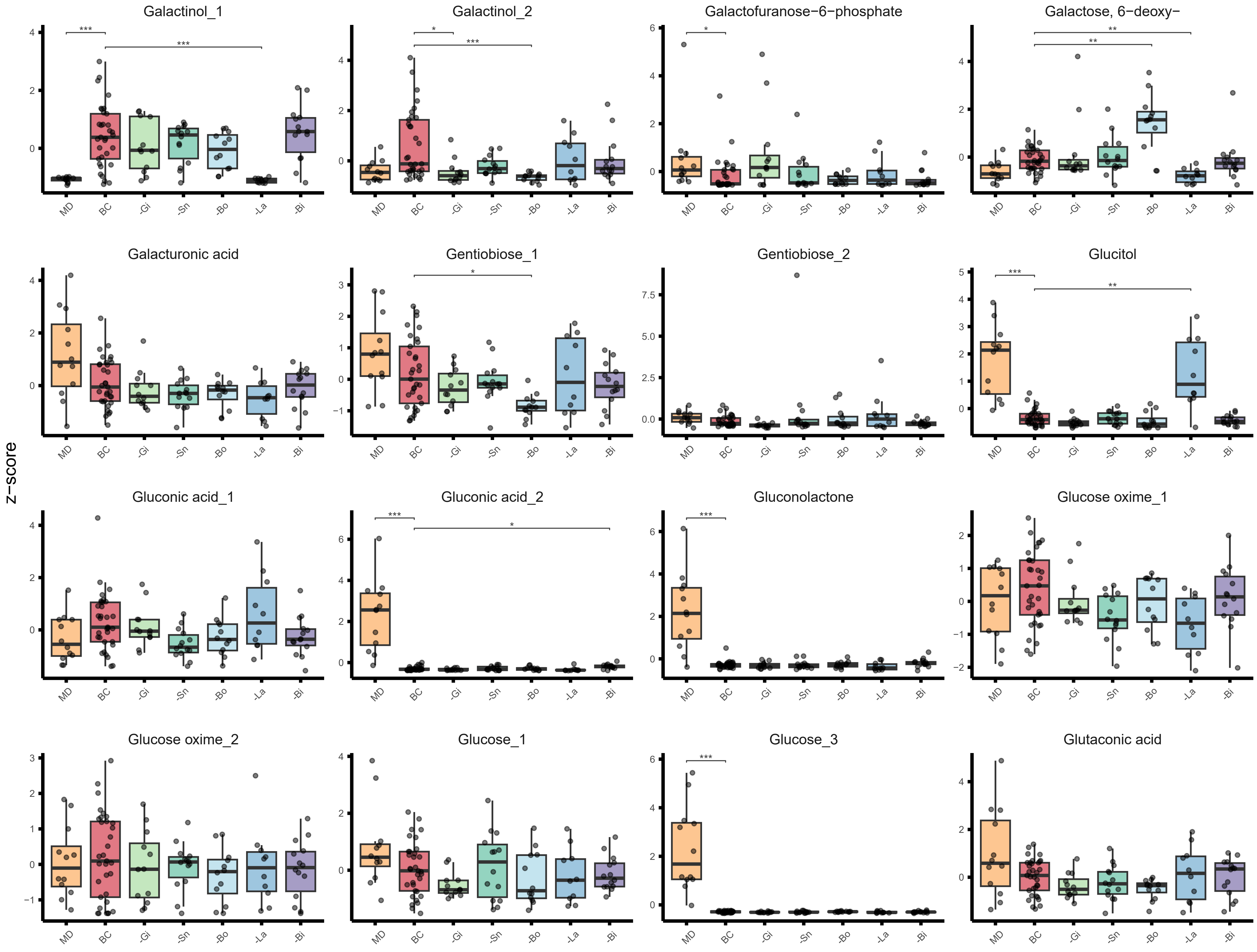

z−score
Galactinol_1
Galactinol_2
Galactofuranose−6−phosphate
Galactose, 6−deoxy−
Galacturonic acid
Gentiobiose_1
Gentiobiose_2
Glucitol
Gluconic acid_1
Gluconic acid_2
Gluconolactone
Glucose oxime_1
Glucose oxime_2
Glucose_1
Glucose_3
Glutaconic acid
MD
BC
-Gi
-Sn
-Bo
-La
-Bi

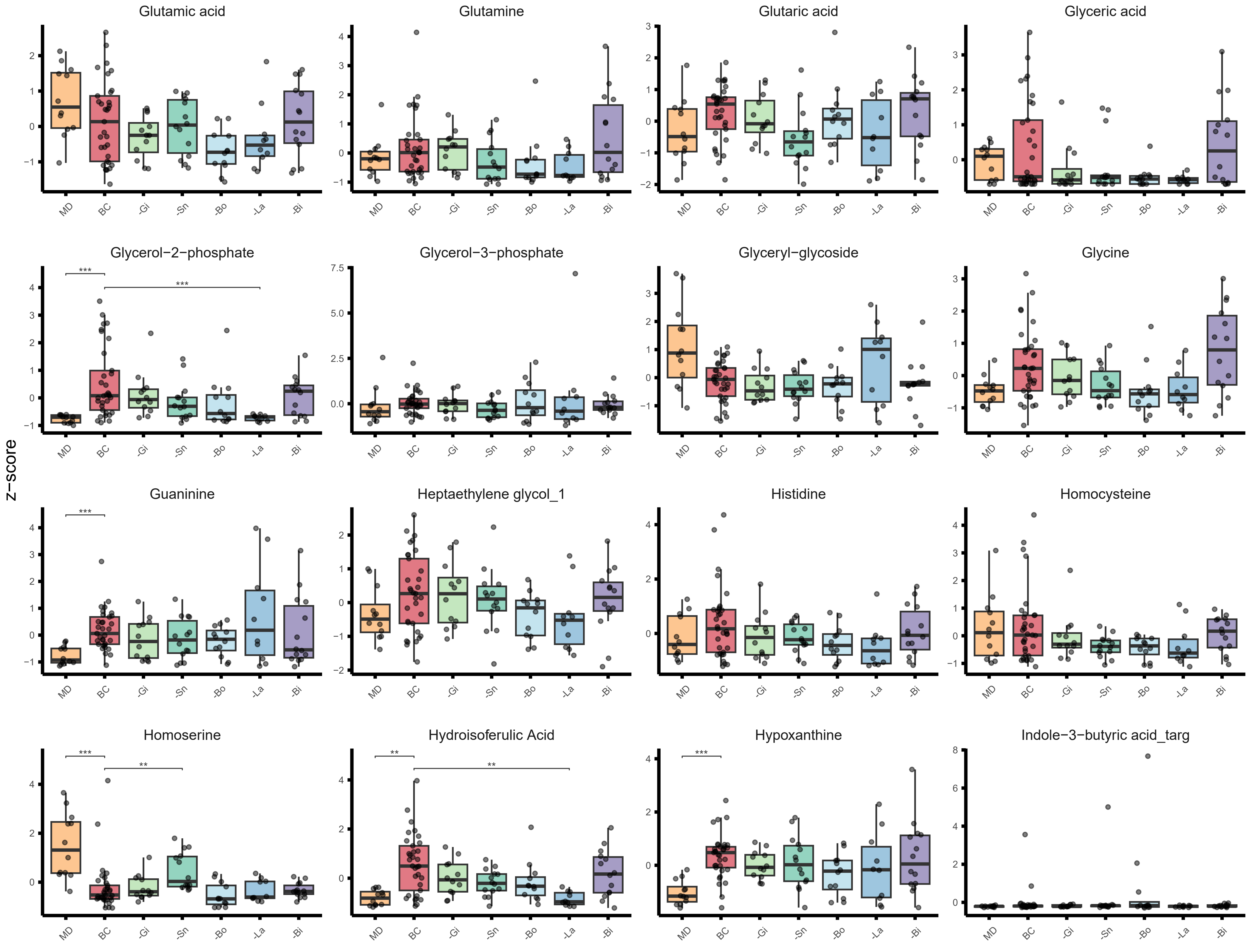

z−score
Glutamic acid
Glutamine
Glutaric acid
Glyceric acid
Glycerol−2−phosphate
Glycerol−3−phosphate
Glyceryl−glycoside
Glycine
Guaninine
Heptaethylene glycol_1
Histidine
Homocysteine
Homoserine
Hydroisoferulic Acid
Hypoxanthine
Indole−3−butyric acid_targ
MD
BC
-Gi
-Sn
-Bo
-La
-Bi
***
**

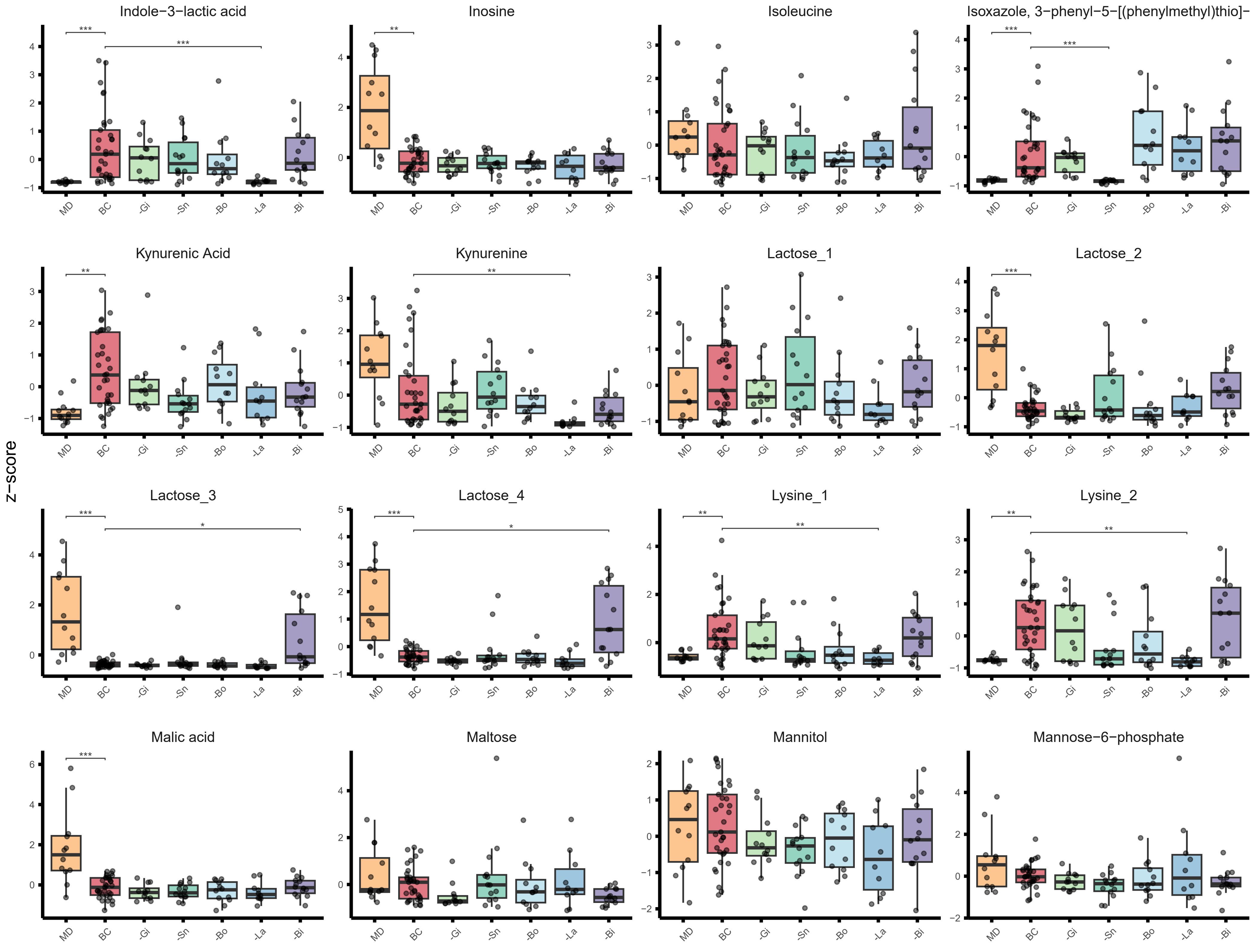
z−score
Indole−3−lactic acid
Inosine
Isoleucine
Isoxazole, 3−phenyl−5−[(phenylmethyl)thio]−
Kynurenic Acid
Kynurenine
Lactose_1
Lactose_2
Lactose_3
Lactose_4
Lysine_1
Lysine_2
Malic acid
Maltose
Mannitol
Mannose−6−phosphate
MD
BC
-Gi
-Sn
-Bo
-La
-Bi

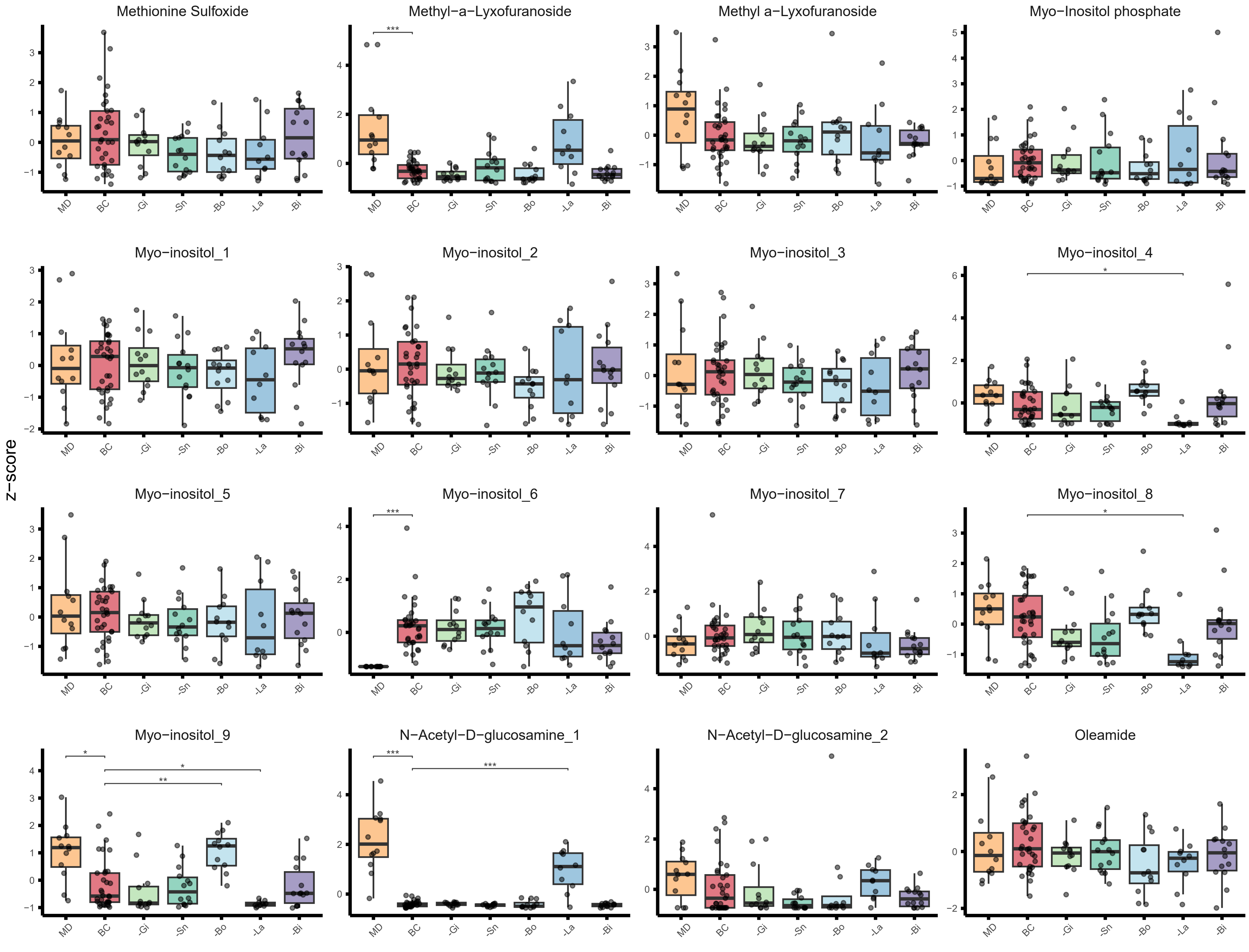

z−score
Methionine Sulfoxide
Methyl−a−Lyxofuranoside
Methyl a−Lyxofuranoside
Myo−Inositol phosphate
Myo−inositol_1
Myo−inositol_2
Myo−inositol_3
Myo−inositol_4
Myo−inositol_5
Myo−inositol_6
Myo−inositol_7
Myo−inositol_8
Myo−inositol_9
N−Acetyl−D−glucosamine_1
N−Acetyl−D−glucosamine_2
Oleamide
MD
BC
-Gi
-Sn
-Bo
-La
-Bi

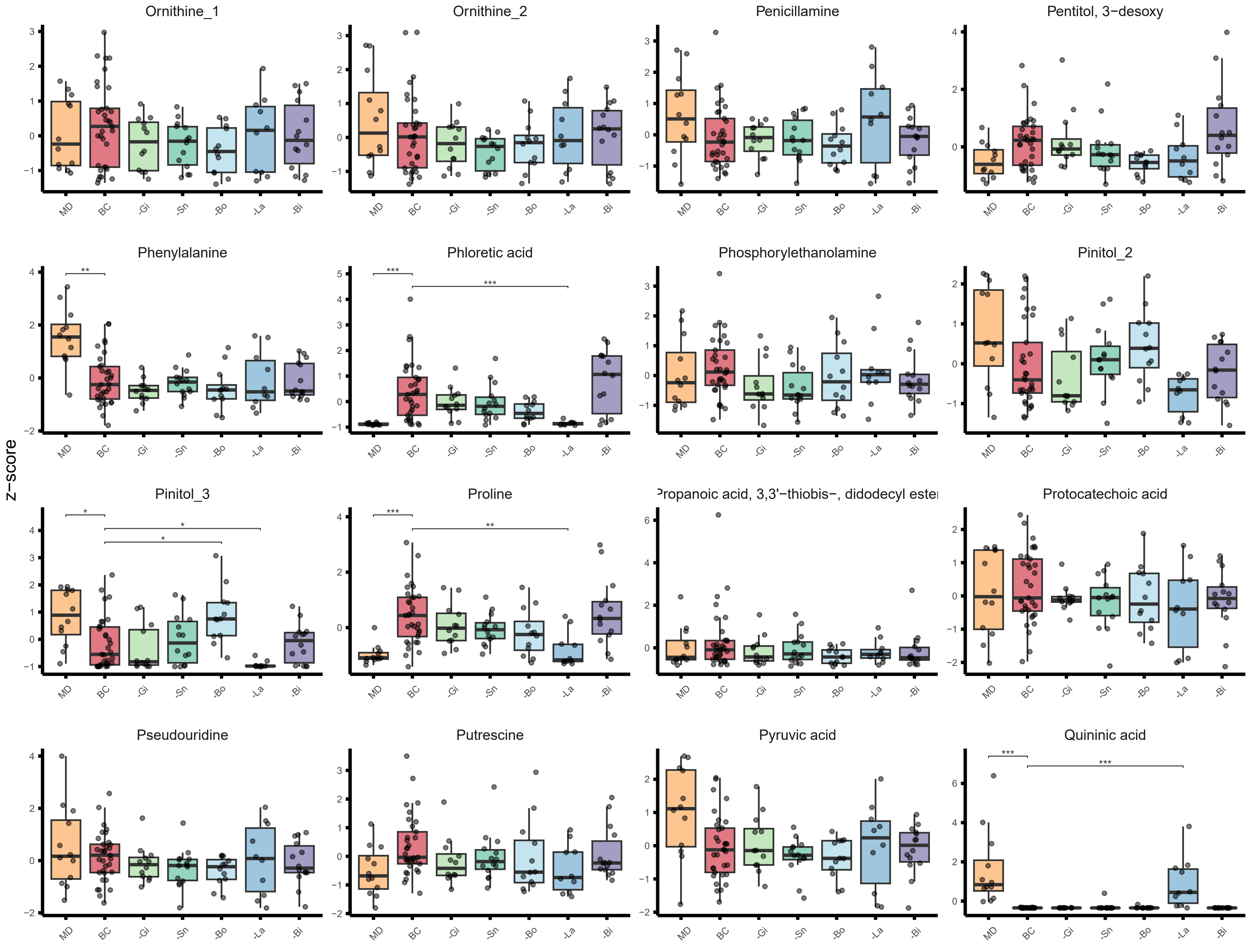
Ornithine_1
Ornithine_2
Penicillamine
Pentitol, 3−desoxy
Phenylalanine
Phloretic acid
Phosphorylethanolamine
Pinitol_2
Pinitol_3
Proline
Propanoic acid, 3,3'−thiobis−, didodecyl ester
Protocatechoic acid
Pseudouridine
Putrescine
Pyruvic acid
Quininic acid
z−score
MD
BC
-Gi
-Sn
-Bo
-La
-Bi

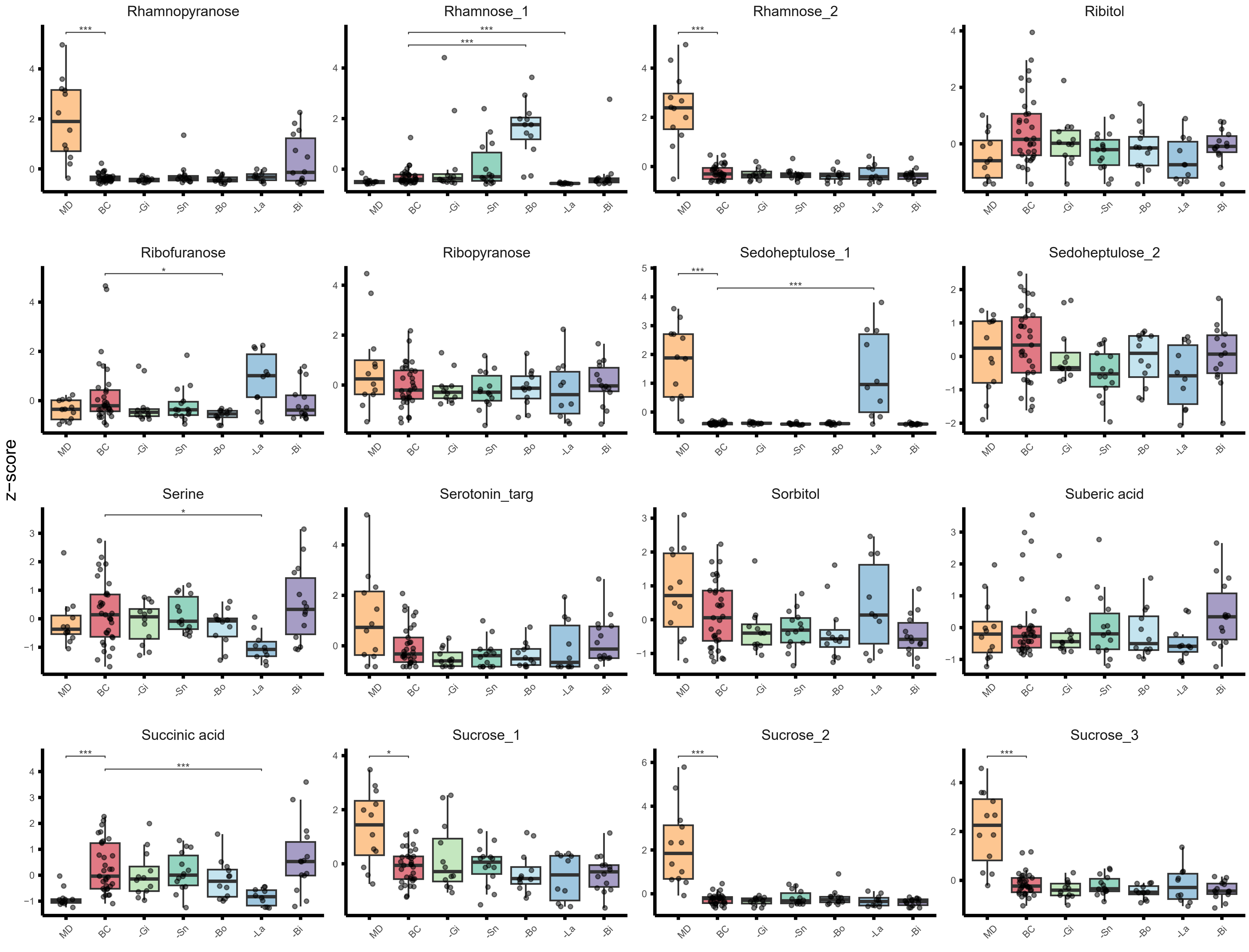

Rhamnopyranose
Rhamnose_1
Rhamnose_2
Ribitol
Ribofuranose
Ribopyranose
Sedoheptulose_1
Sedoheptulose_2
Serine
Serotonin_targ
Sorbitol
Suberic acid
Succinic acid
Sucrose_1
Sucrose_2
Sucrose_3
z-score
MD
BC
-Gi
-Sn
-Bo
-La
-Bi
***
*

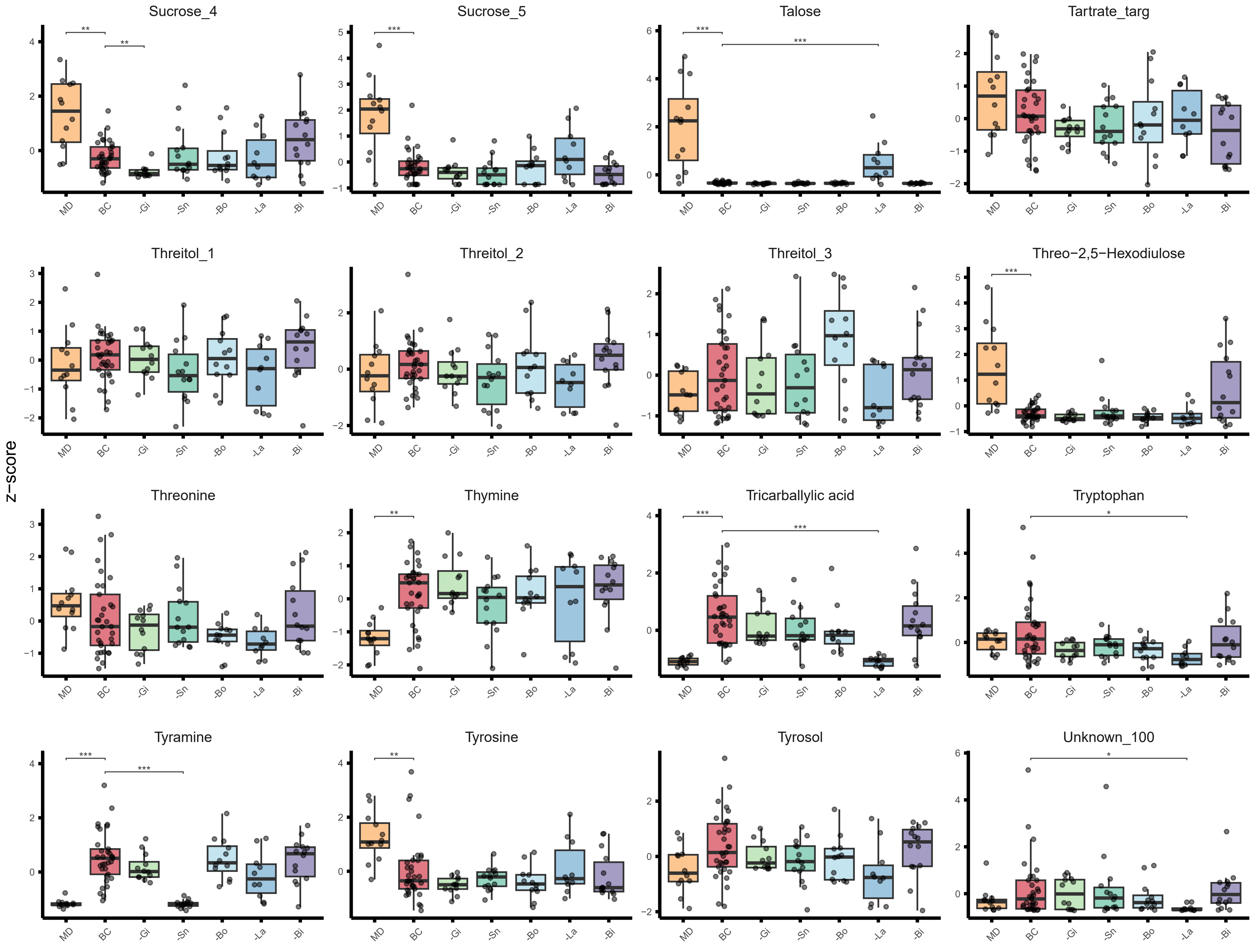
Sucrose_4
Sucrose_5
Talose
Tartrate_targ
Threitol_1
Threitol_2
Threitol_3
Threo−2,5−Hexodiulose
Threonine
Thymine
Tricarballylic acid
Tryptophan
Tyramine
Tyrosine
Tyrosol
Unknown_100
z−score
MD
BC
-Gi
-Sn
-Bo
-La
-Bi

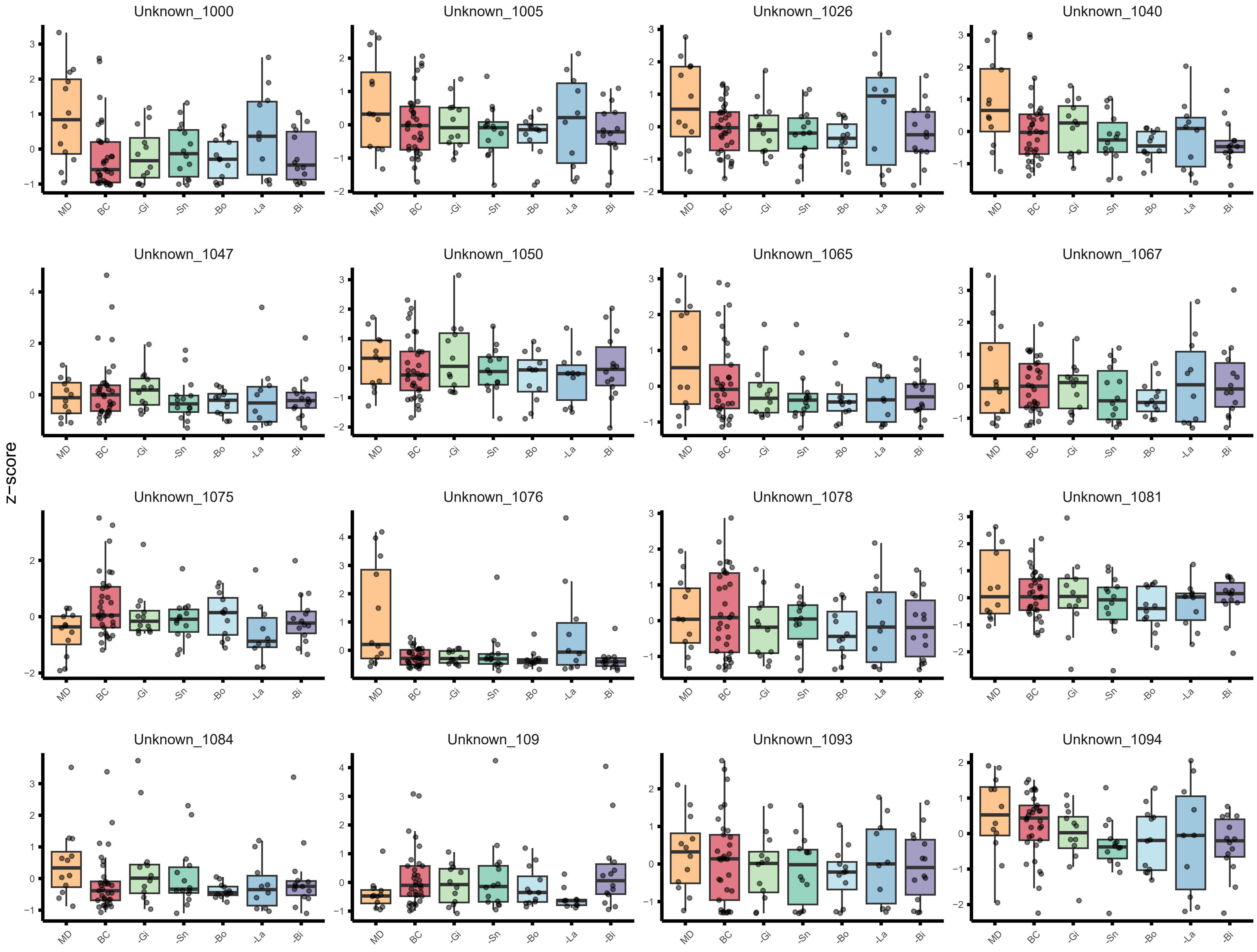

z−score
Unknown_1000
Unknown_1005
Unknown_1026
Unknown_1040
Unknown_1047
Unknown_1050
Unknown_1065
Unknown_1067
Unknown_1075
Unknown_1076
Unknown_1078
Unknown_1081
Unknown_1084
Unknown_109
Unknown_1093
Unknown_1094
MD
BC
-Gi
-Sn
-Bo
-La
-Bi

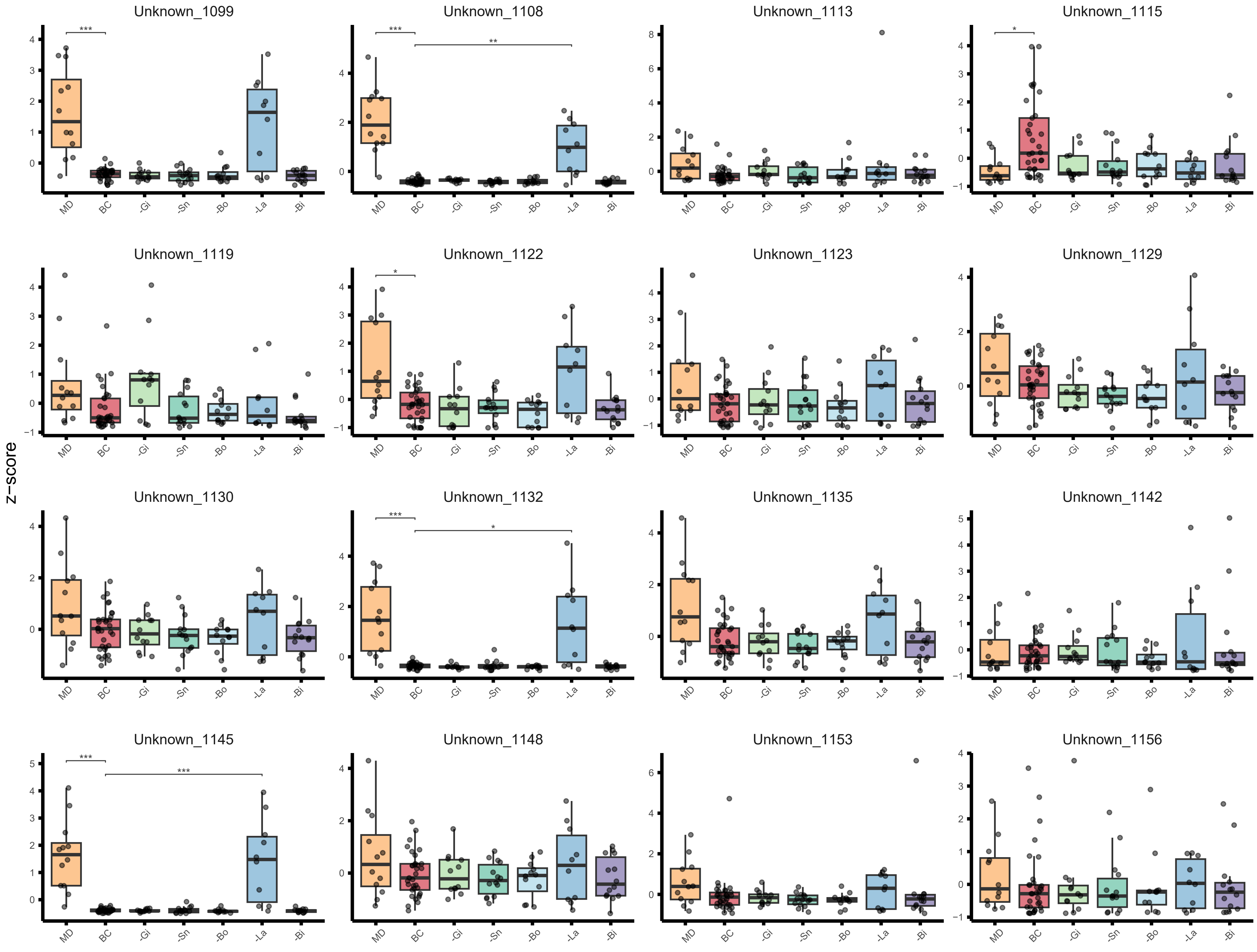

z−score
Unknown_1099
Unknown_1108
Unknown_1113
Unknown_1115
Unknown_1119
Unknown_1122
Unknown_1123
Unknown_1129
Unknown_1130
Unknown_1132
Unknown_1135
Unknown_1142
Unknown_1145
Unknown_1148
Unknown_1153
Unknown_1156
MD
BC
-Gi
-Sn
-Bo
-La
-Bi

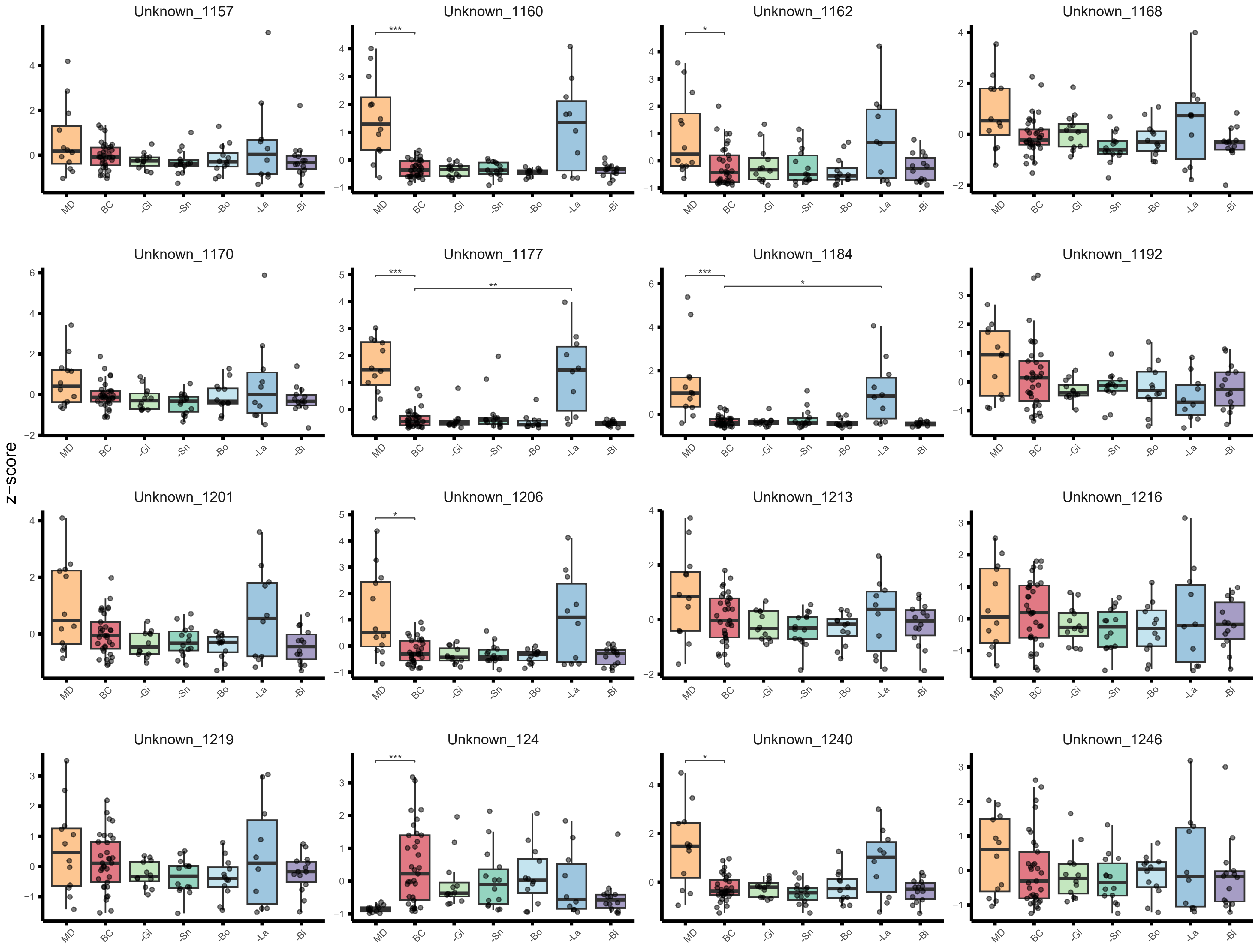

z−score
Unknown_1157
Unknown_1160
Unknown_1162
Unknown_1168
Unknown_1170
Unknown_1177
Unknown_1184
Unknown_1192
Unknown_1201
Unknown_1206
Unknown_1213
Unknown_1216
Unknown_1219
Unknown_124
Unknown_1240
Unknown_1246
MD
BC
-Gi
-Sn
-Bo
-La
-Bi

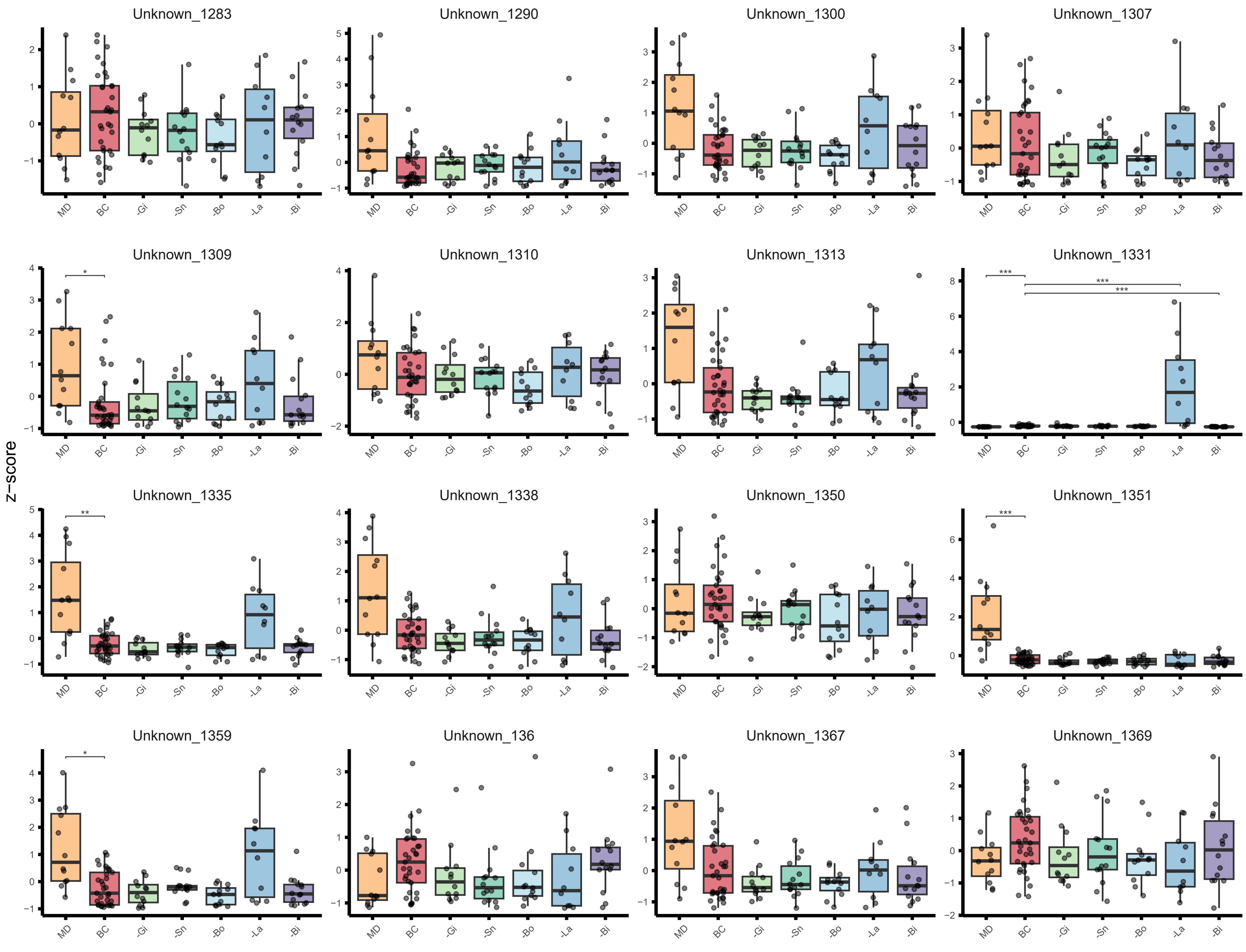

z−score
Unknown_1283
Unknown_1290
Unknown_1300
Unknown_1307
Unknown_1309
Unknown_1310
Unknown_1313
Unknown_1331
Unknown_1335
Unknown_1338
Unknown_1350
Unknown_1351
Unknown_1359
Unknown_136
Unknown_1367
Unknown_1369
MD
BC
-Gi
-Sn
-Bo
-La
-Bi

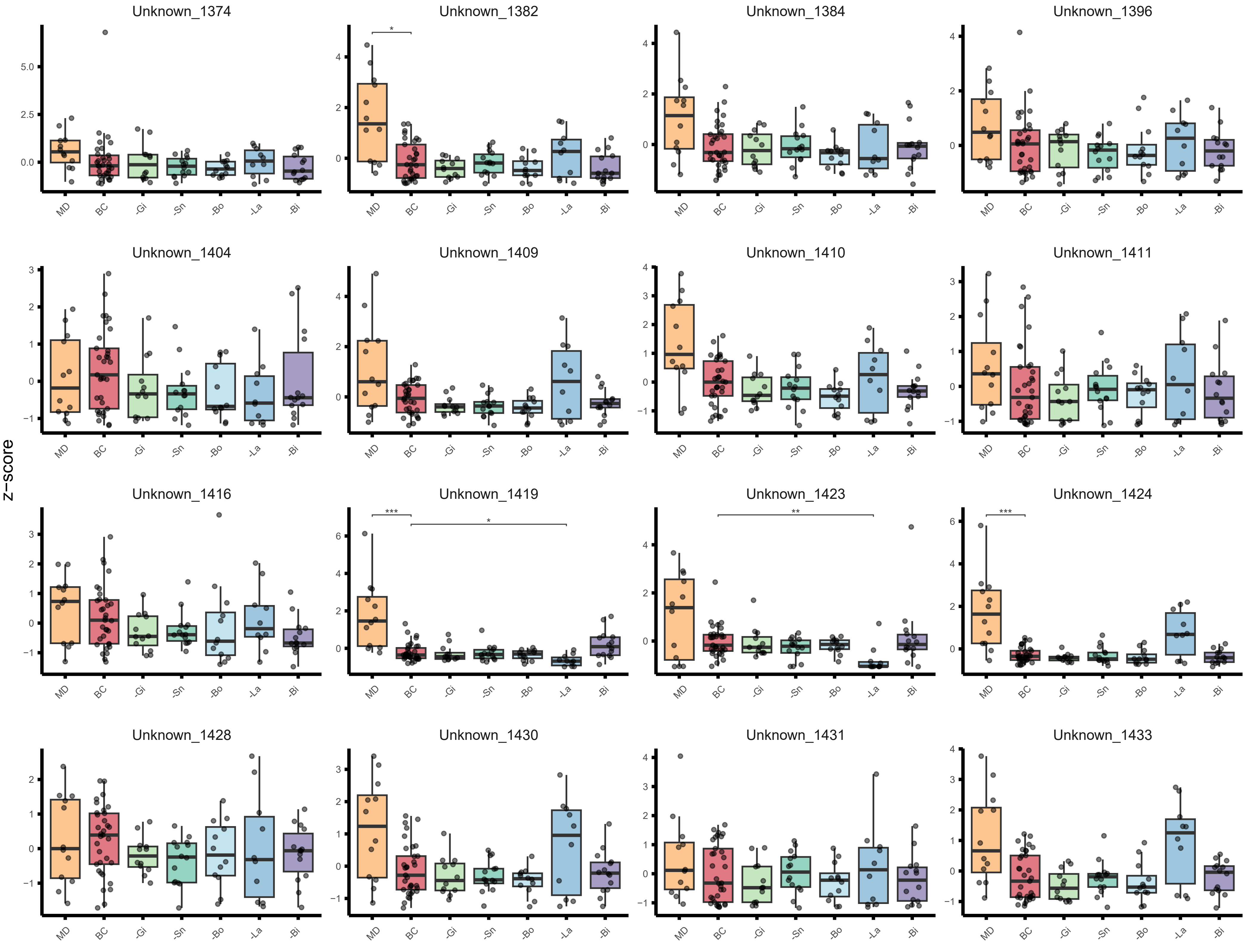

Unknown_1374
Unknown_1382
*
Unknown_1384
Unknown_1396
Unknown_1404
Unknown_1409
Unknown_1410
Unknown_1411
Unknown_1416
Unknown_1419
***
*
Unknown_1423
**
Unknown_1424
***
Unknown_1428
Unknown_1430
Unknown_1431
Unknown_1433
z−score
MD
BC
-Gi
-Sn
-Bo
-La
-Bi

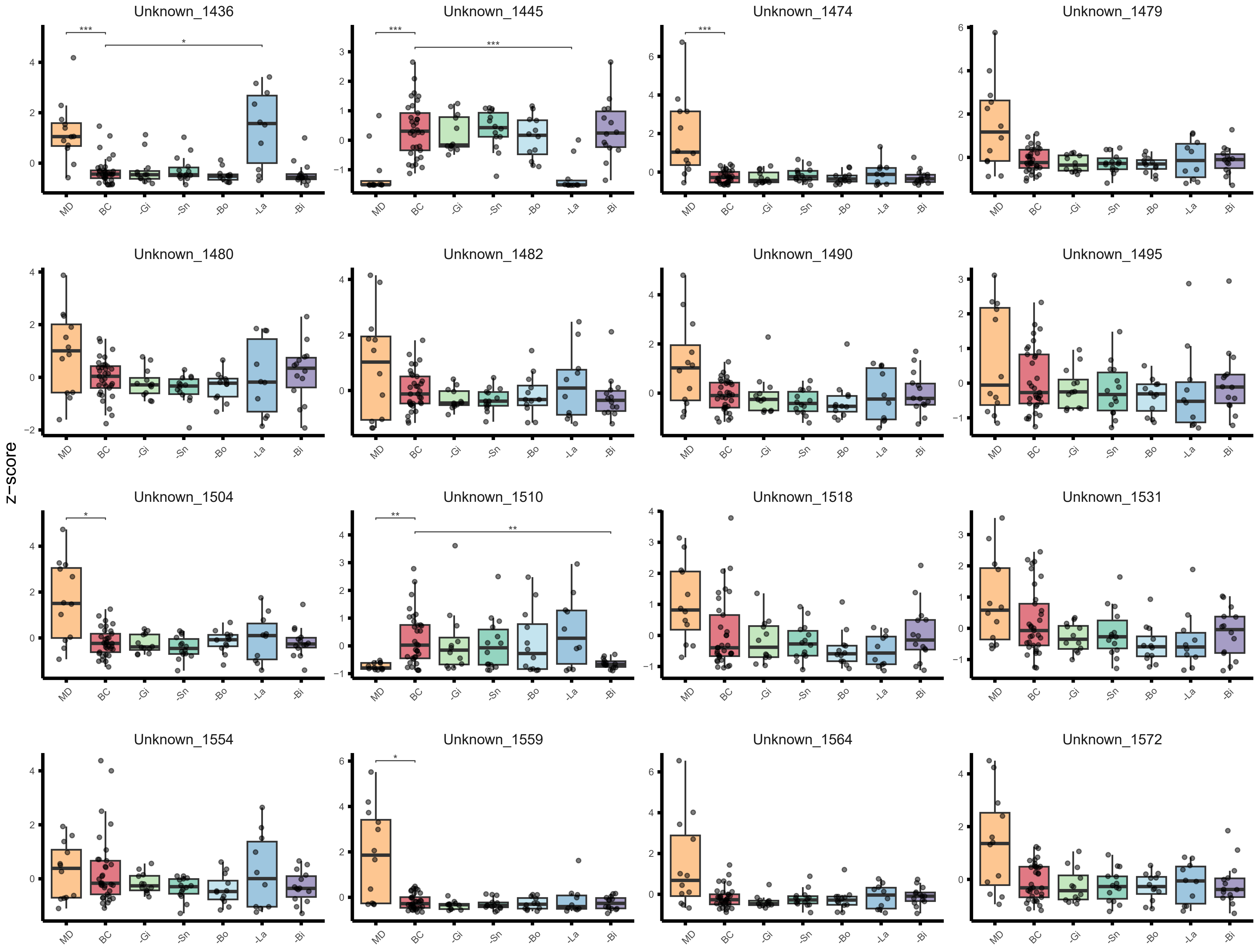

Unknown_1436
Unknown_1445
Unknown_1474
Unknown_1479
Unknown_1480
Unknown_1482
Unknown_1490
Unknown_1495
Unknown_1504
Unknown_1510
Unknown_1518
Unknown_1531
Unknown_1554
Unknown_1559
Unknown_1564
Unknown_1572
z−score
MD
BC
-Gi
-Sn
-Bo
-La
-Bi

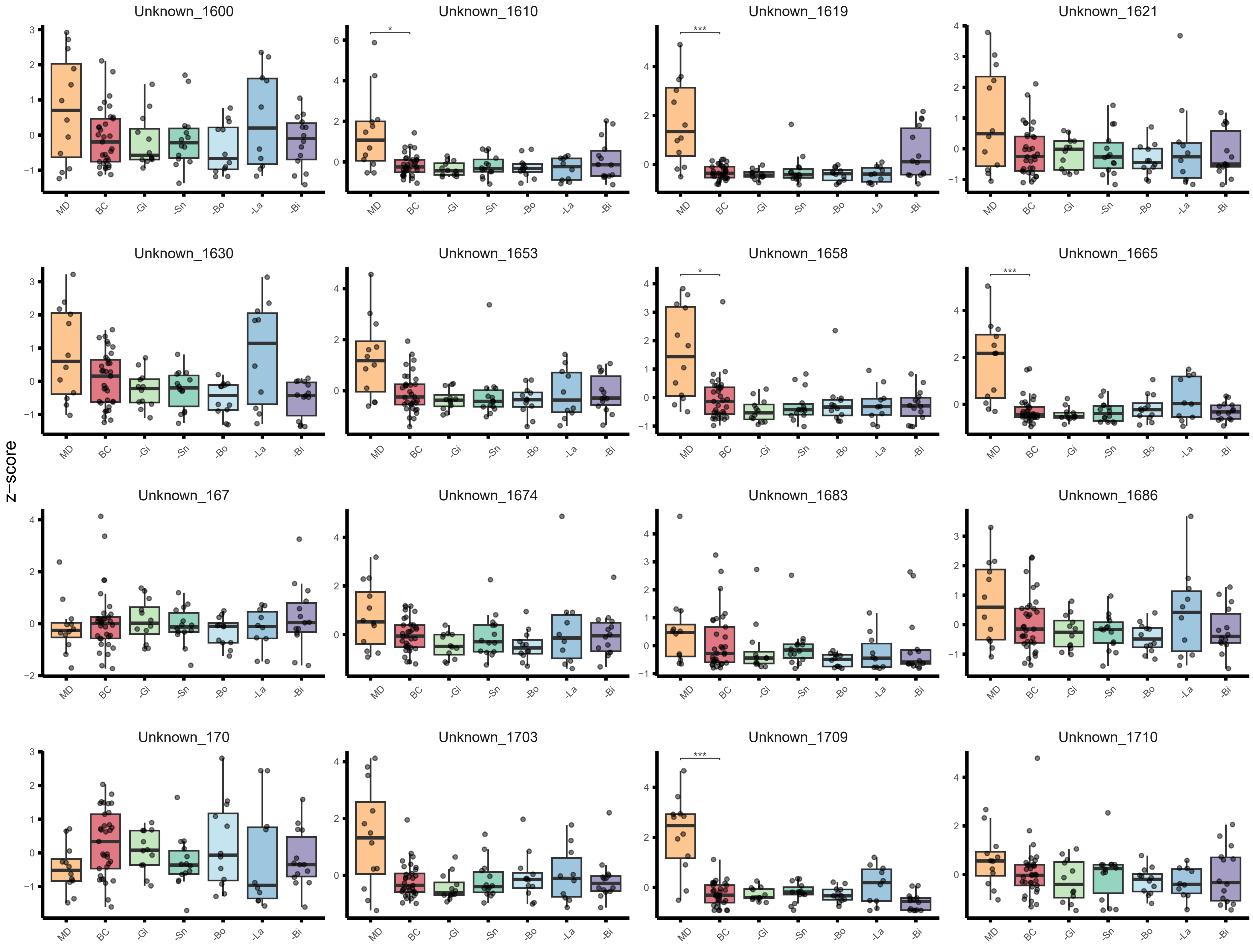
z−score
Unknown_1600
Unknown_1610
Unknown_1619
Unknown_1621
Unknown_1630
Unknown_1653
Unknown_1658
Unknown_1665
Unknown_167
Unknown_1674
Unknown_1683
Unknown_1686
Unknown_170
Unknown_1703
Unknown_1709
Unknown_1710
MD
BC
-Gi
-Sn
-Bo
-La
-Bi
*
***

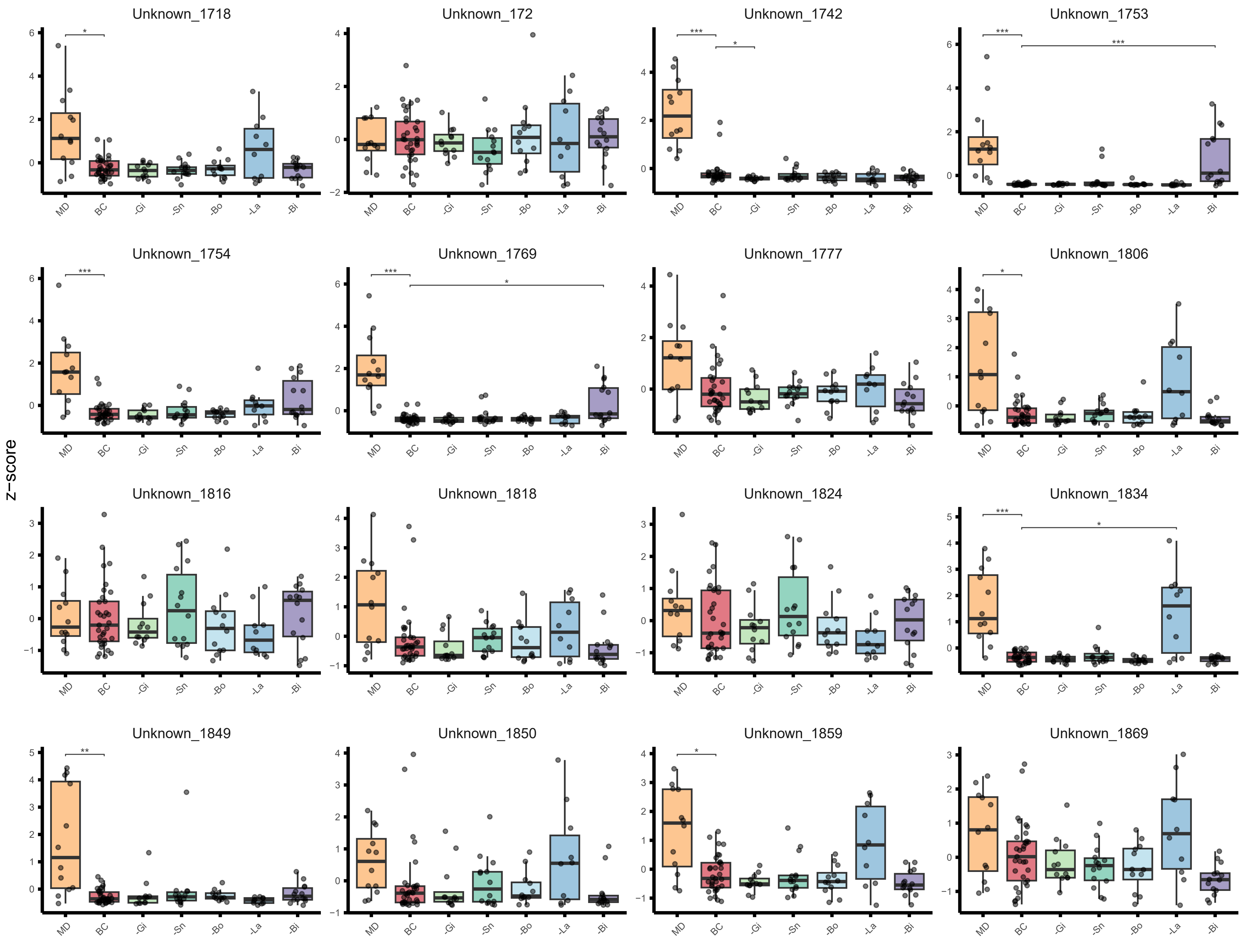

z−score
Unknown_1718
Unknown_172
Unknown_1742
Unknown_1753
Unknown_1754
Unknown_1769
Unknown_1777
Unknown_1806
Unknown_1816
Unknown_1818
Unknown_1824
Unknown_1834
Unknown_1849
Unknown_1850
Unknown_1859
Unknown_1869
MD
BC
-Gi
-Sn
-Bo
-La
-Bi

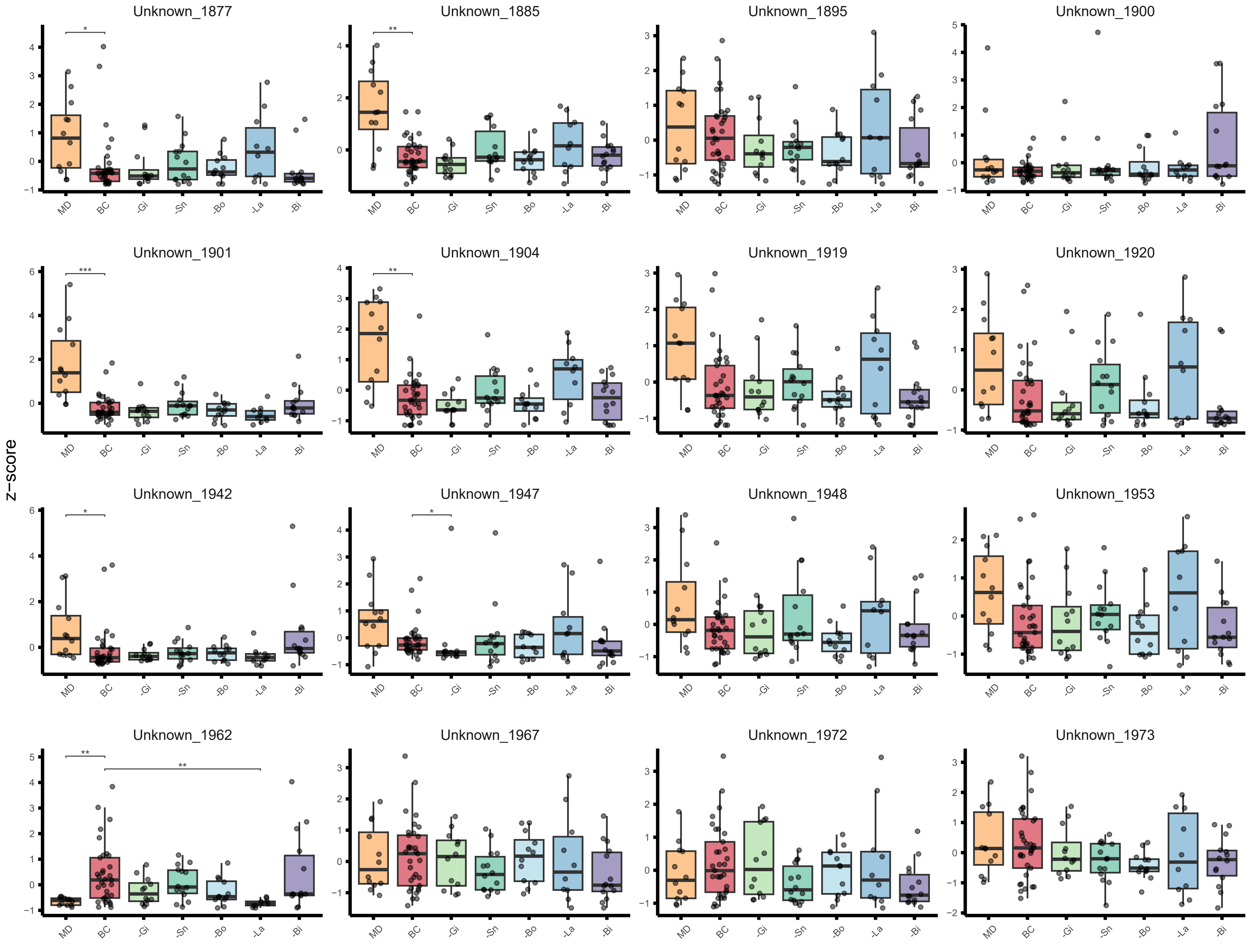
z−score
Unknown_1877
Unknown_1885
Unknown_1895
Unknown_1900
Unknown_1901
Unknown_1904
Unknown_1919
Unknown_1920
Unknown_1942
Unknown_1947
Unknown_1948
Unknown_1953
Unknown_1962
Unknown_1967
Unknown_1972
Unknown_1973
MD
BC
-Gi
-Sn
-Bo
-La
-Bi

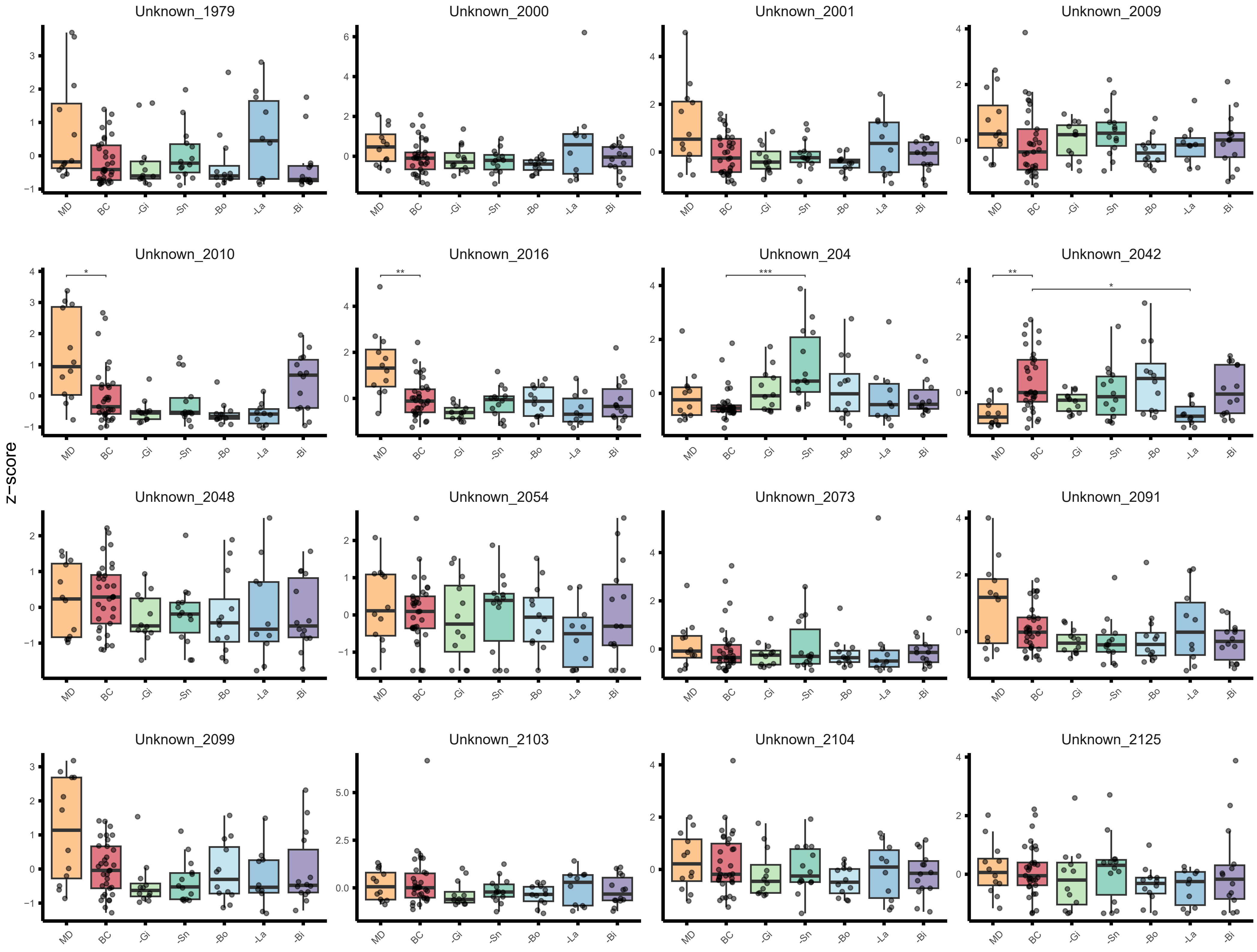

z−score
Unknown_1979
Unknown_2000
Unknown_2001
Unknown_2009
Unknown_2010
Unknown_2016
Unknown_204
Unknown_2042
Unknown_2048
Unknown_2054
Unknown_2073
Unknown_2091
Unknown_2099
Unknown_2103
Unknown_2104
Unknown_2125
MD
BC
-Gi
-Sn
-Bo
-La
-Bi

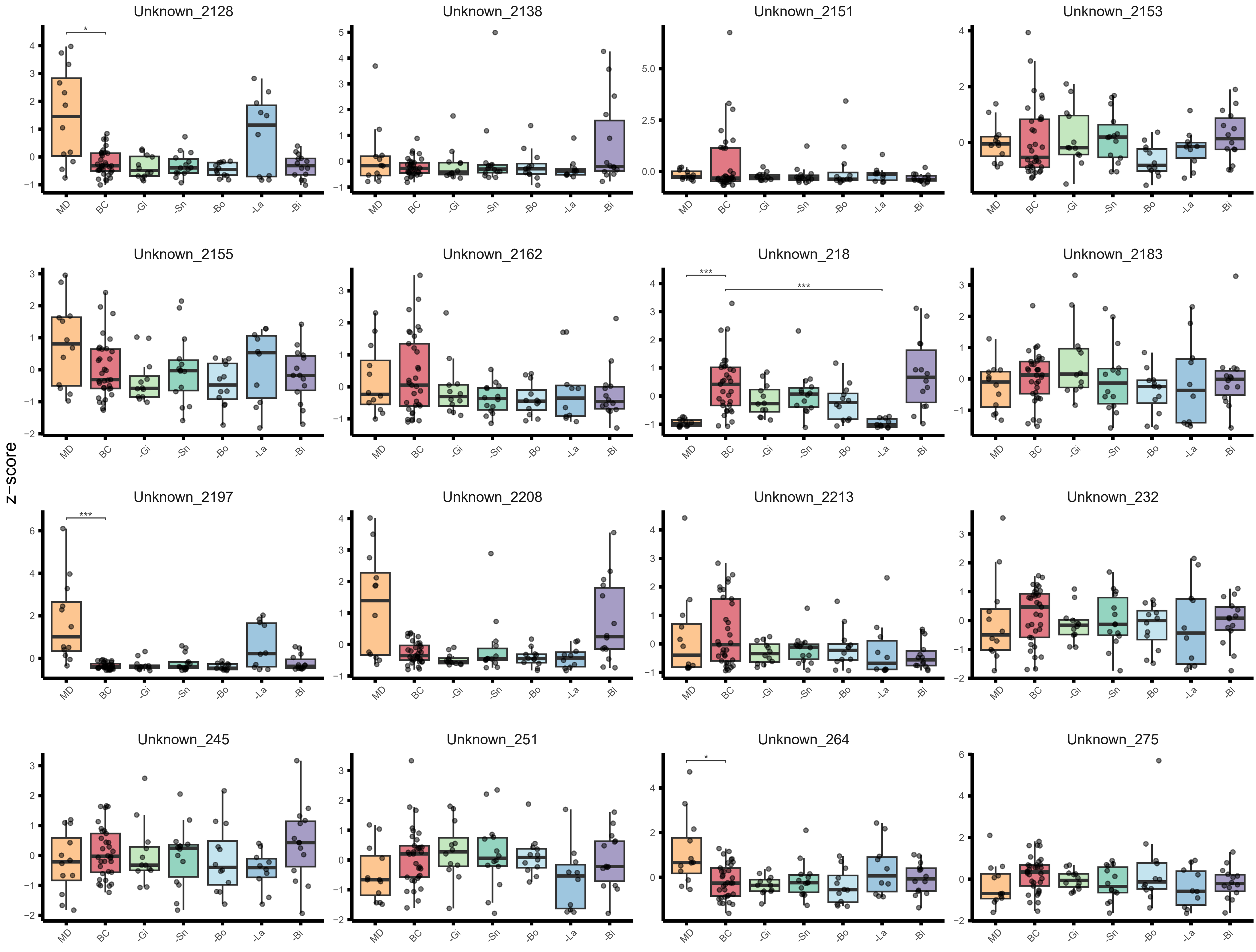

Unknown_2128
Unknown_2138
Unknown_2151
Unknown_2153
Unknown_2155
Unknown_2162
Unknown_218
Unknown_2183
Unknown_2197
Unknown_2208
Unknown_2213
Unknown_232
Unknown_245
Unknown_251
Unknown_264
Unknown_275
z−score
MD
BC
-Gi
-Sn
-Bo
-La
-Bi

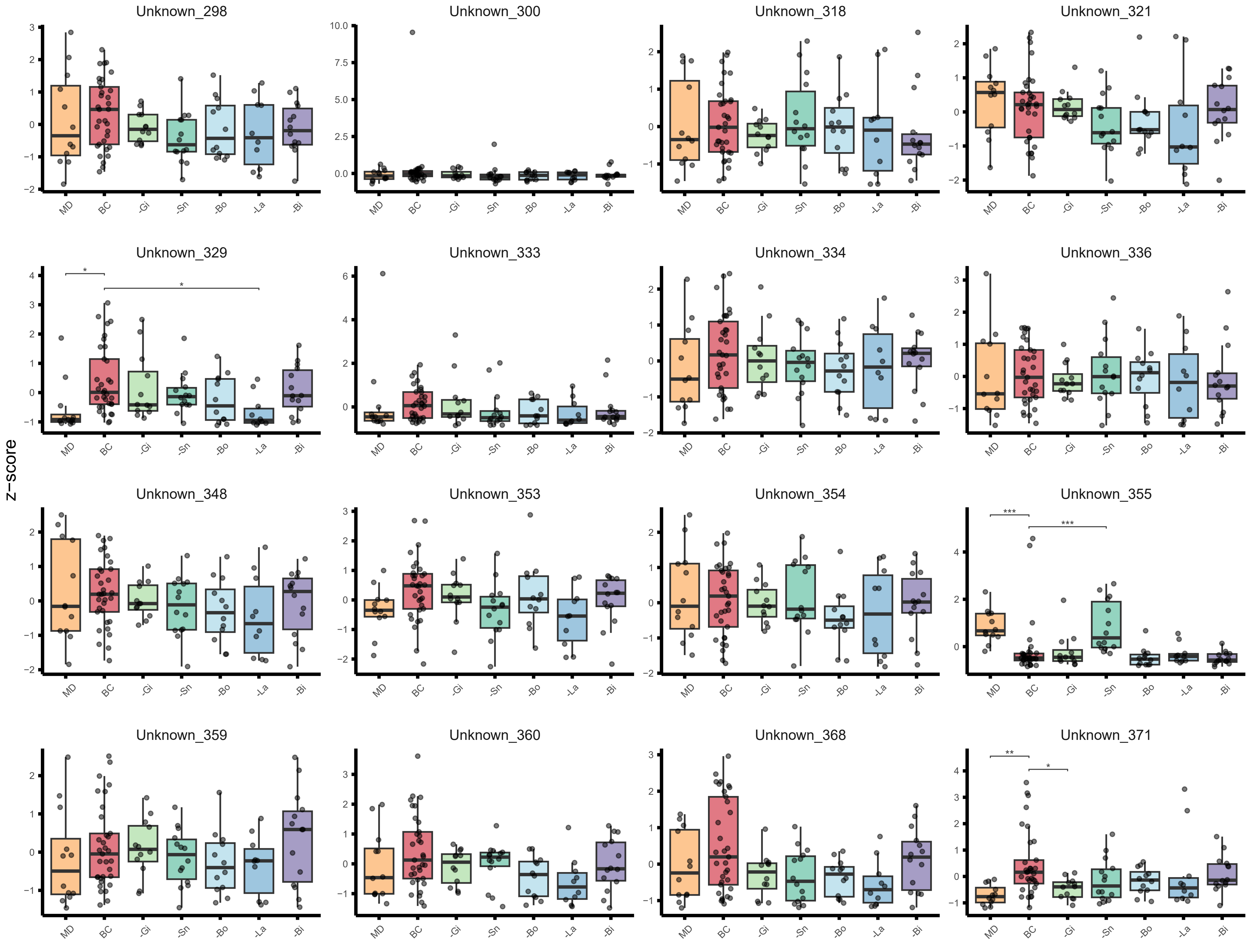
Unknown_298
Unknown_300
Unknown_318
Unknown_321
Unknown_329
Unknown_333
Unknown_334
Unknown_336
Unknown_348
Unknown_353
Unknown_354
Unknown_355
Unknown_359
Unknown_360
Unknown_368
Unknown_371
z−score
MD
BC
-Gi
-Sn
-Bo
-La
-Bi

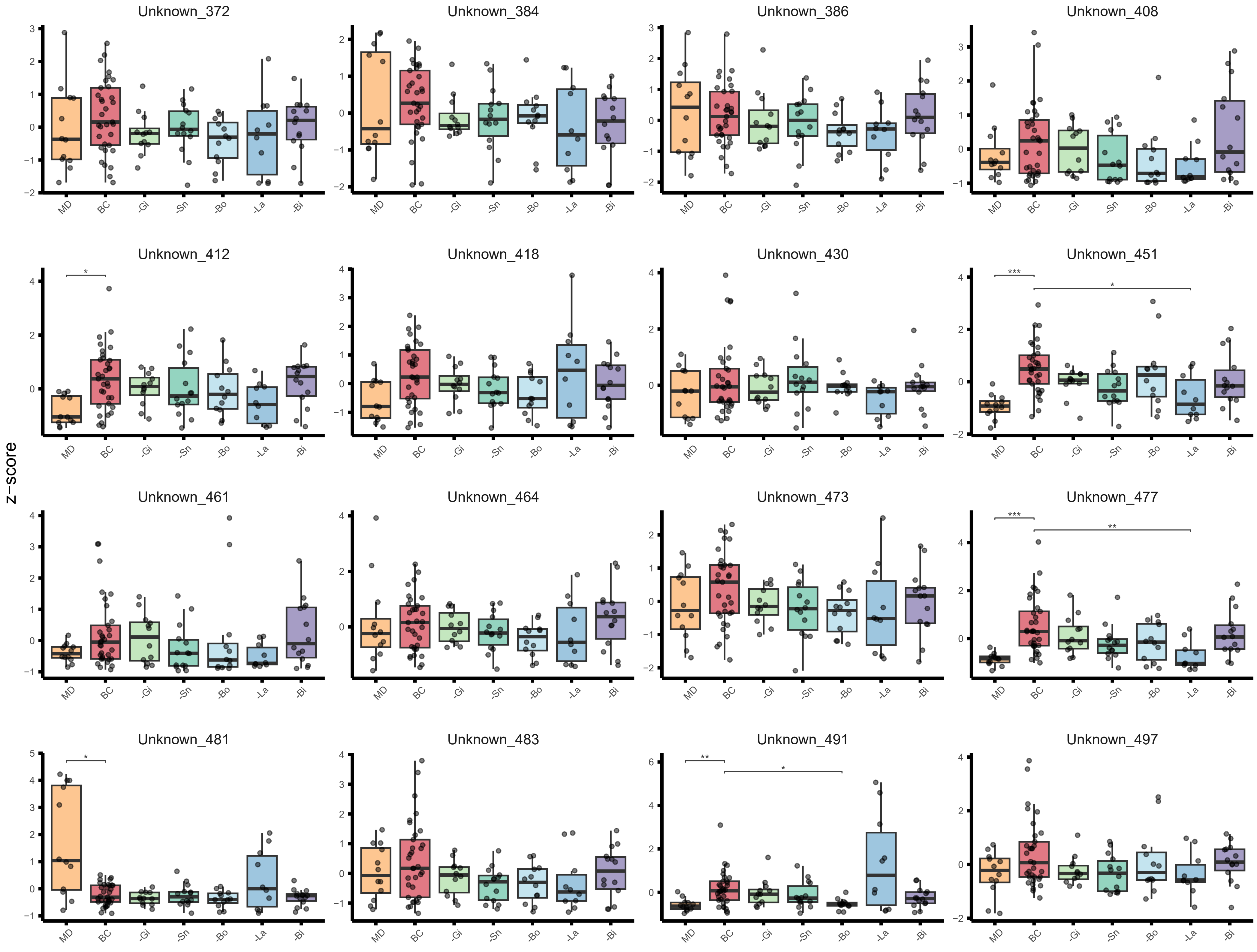
z−score
Unknown_372
Unknown_384
Unknown_386
Unknown_408
Unknown_412
Unknown_418
Unknown_430
Unknown_451
Unknown_461
Unknown_464
Unknown_473
Unknown_477
Unknown_481
Unknown_483
Unknown_491
Unknown_497
MD
BC
-Gi
-Sn
-Bo
-La
-Bi

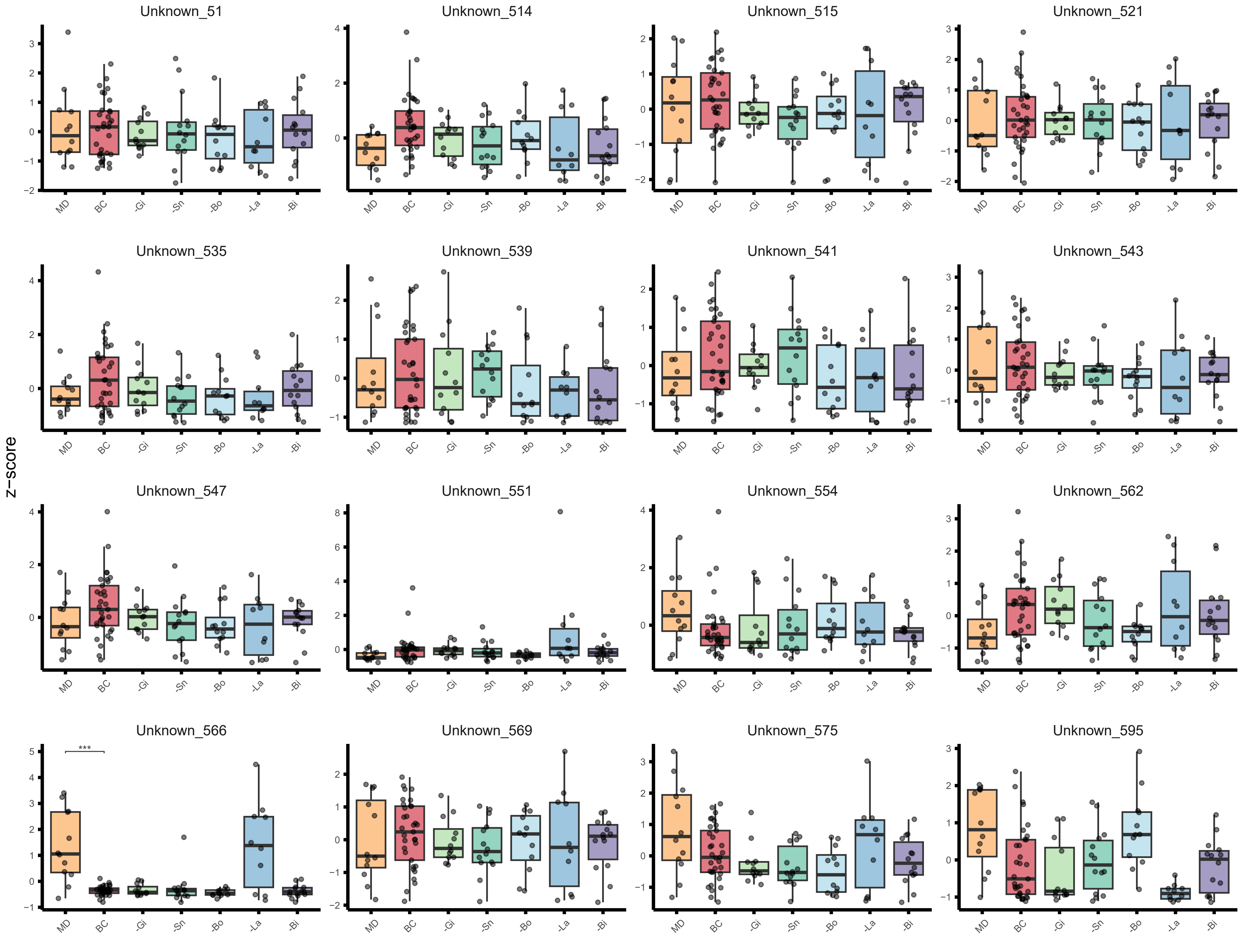

z−score
Unknown_51
Unknown_514
Unknown_515
Unknown_521
Unknown_535
Unknown_539
Unknown_541
Unknown_543
Unknown_547
Unknown_551
Unknown_554
Unknown_562
Unknown_566
Unknown_569
Unknown_575
Unknown_595
***
MD
BC
-Gi
-Sn
-Bo
-La
-Bi

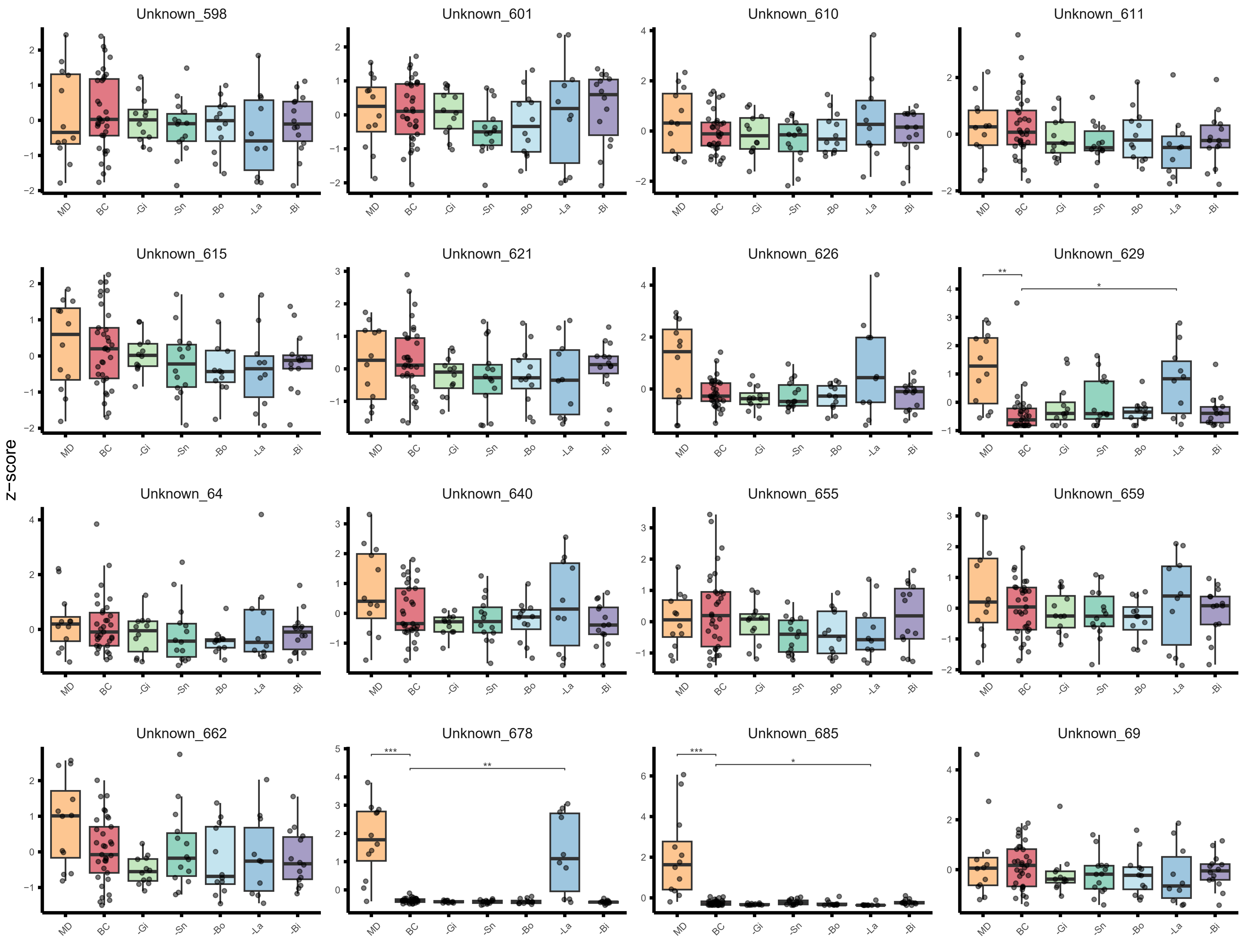

z−score
Unknown_598
Unknown_601
Unknown_610
Unknown_611
Unknown_615
Unknown_621
Unknown_626
Unknown_629
Unknown_64
Unknown_640
Unknown_655
Unknown_659
Unknown_662
Unknown_678
Unknown_685
Unknown_69
MD
BC
-Gi
-Sn
-Bo
-La
-Bi

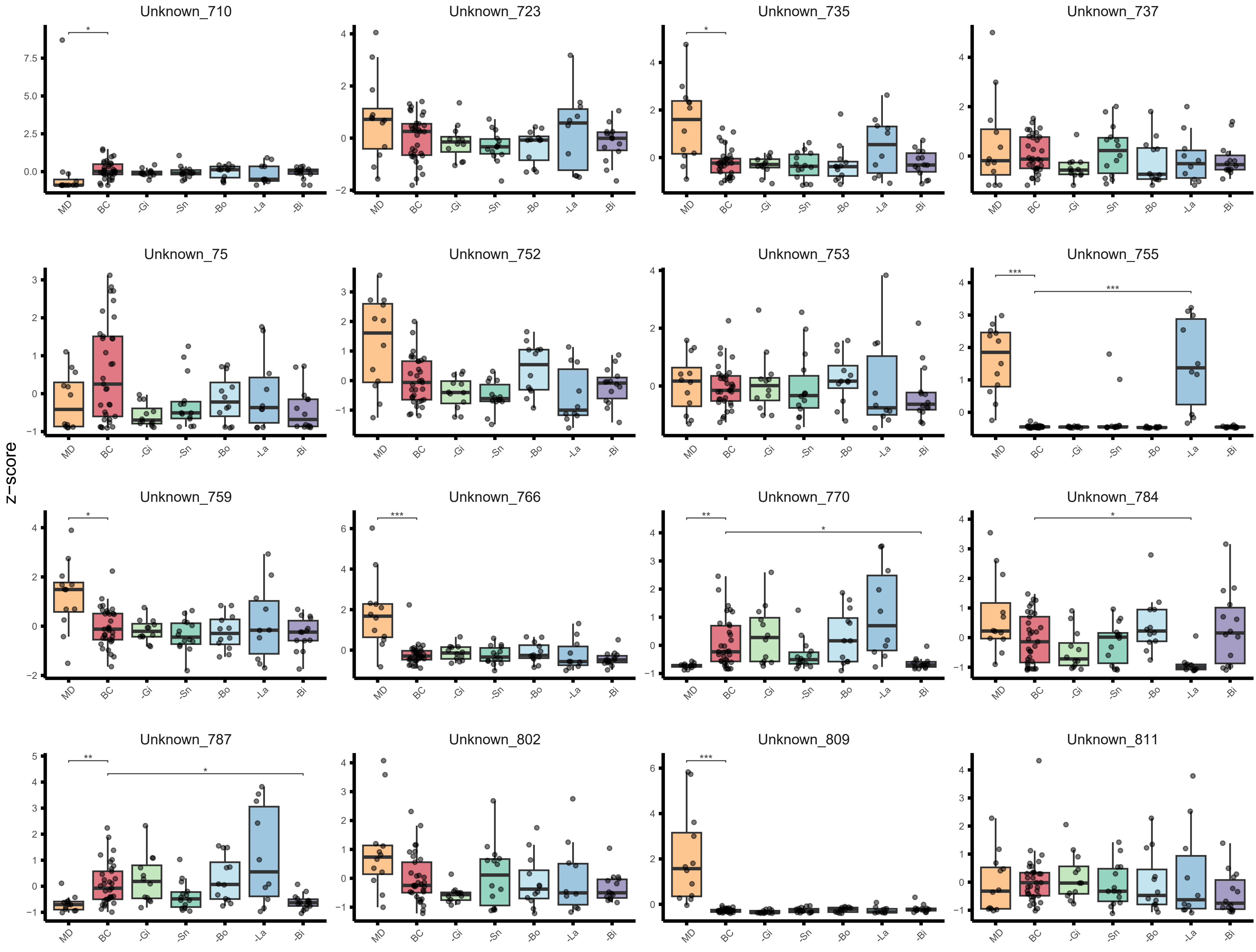

Unknown_710
Unknown_723
Unknown_735
Unknown_737
Unknown_75
Unknown_752
Unknown_753
Unknown_755
Unknown_759
Unknown_766
Unknown_770
Unknown_784
Unknown_787
Unknown_802
Unknown_809
Unknown_811
z-score
MD
BC
-Gi
-Sn
-Bo
-La
-Bi

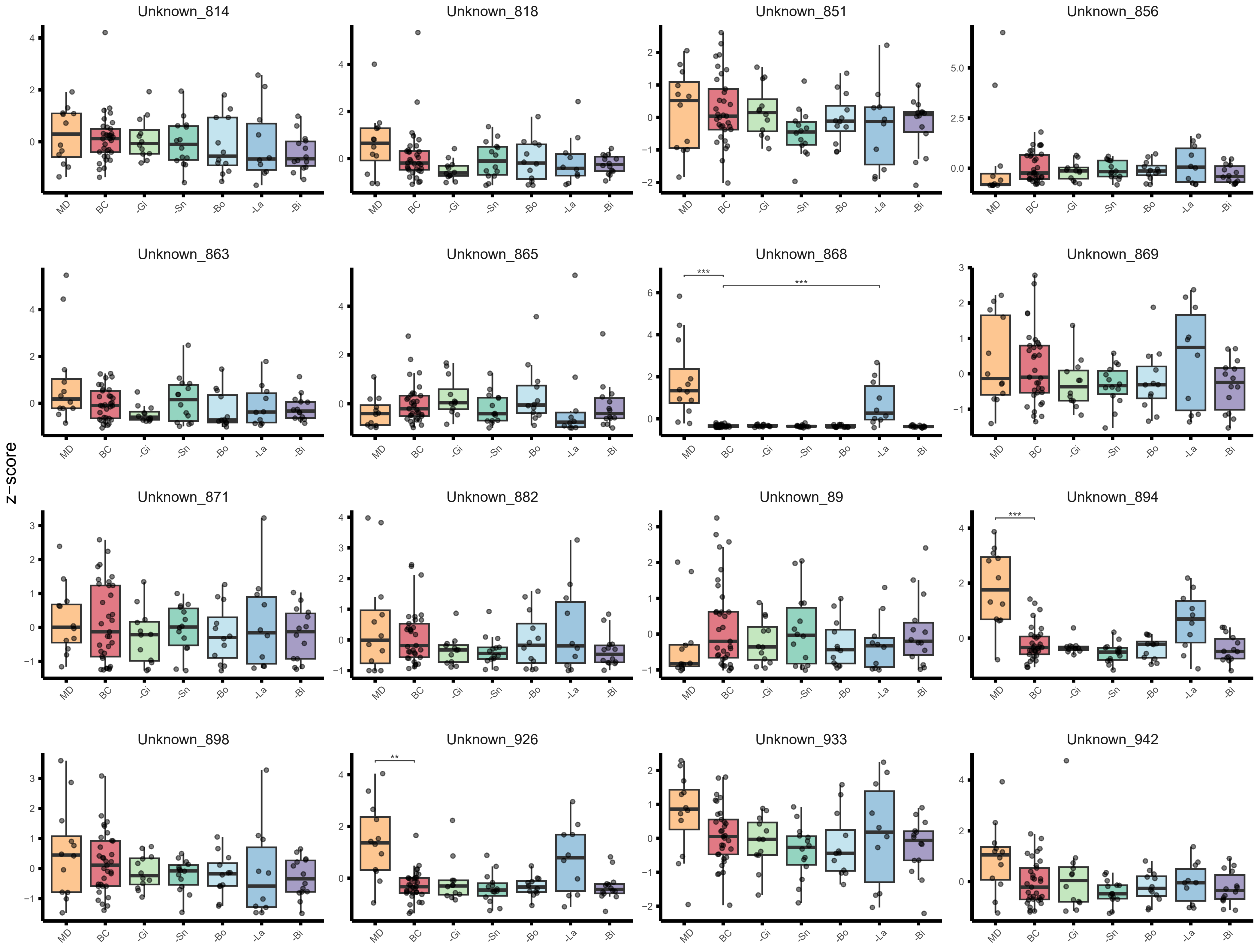

z−score
Unknown_814
Unknown_818
Unknown_851
Unknown_856
Unknown_863
Unknown_865
Unknown_868
Unknown_869
Unknown_871
Unknown_882
Unknown_89
Unknown_894
Unknown_898
Unknown_926
Unknown_933
Unknown_942
MD
BC
-Gi
-Sn
-Bo
-La
-Bi
***
**

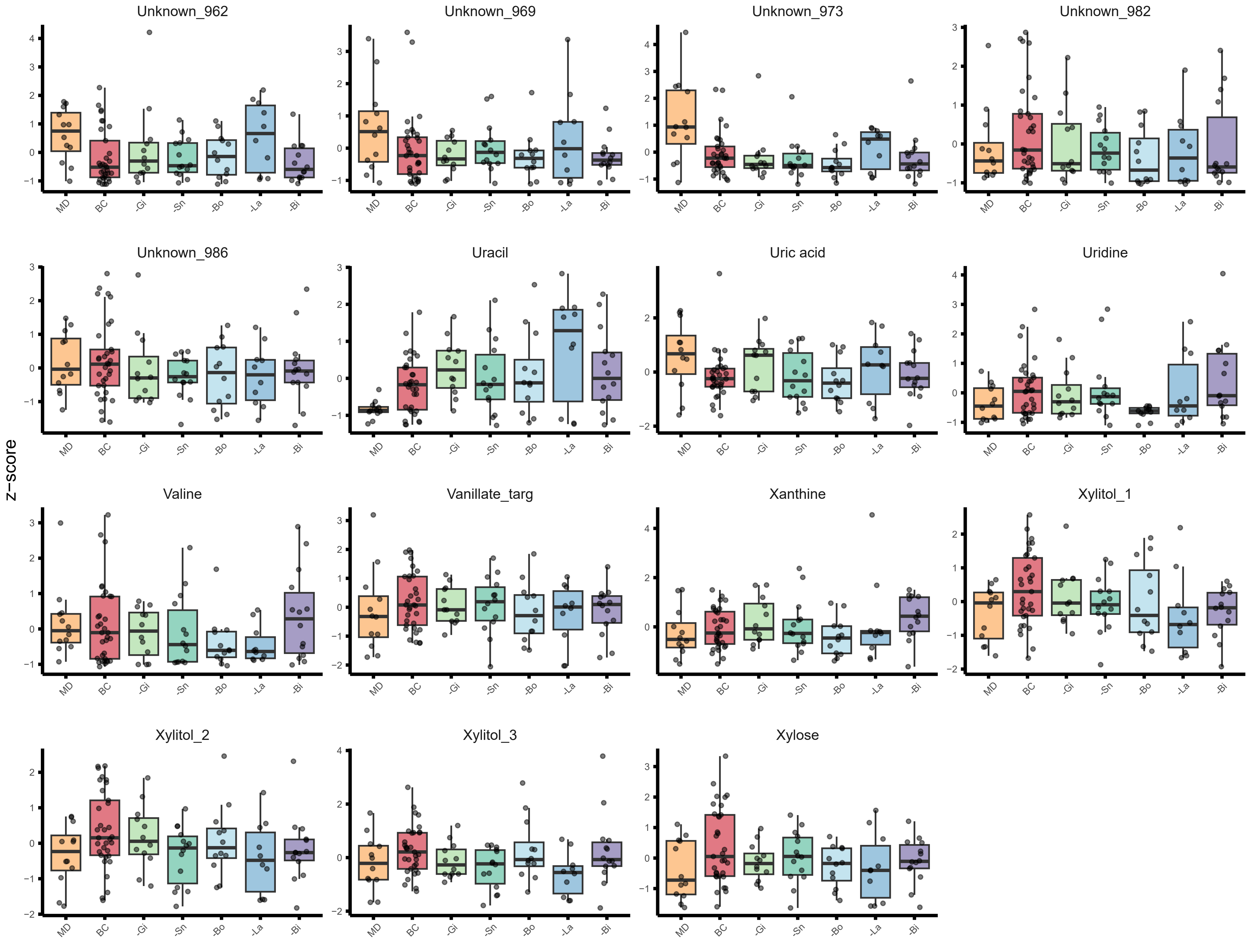

Unknown_962
Unknown_969
Unknown_973
Unknown_982
Unknown_986
Uracil
Uric acid
Uridine
Valine
Vanillate_targ
Xanthine
Xylitol_1
Xylitol_2
Xylitol_3
Xylose
z−score
MD
BC
-Gi
-Sn
-Bo
-La
-Bi
